# Photoactivatable aggregation-induced emission probes for lipid droplets-specific live cell imaging†
†Electronic supplementary information (ESI) available: Structural and photophysical characterization data for **1** and **2**, UV-vis and PL spectra, DFT calculations, cell imaging, cell viability, and NMR spectra (PDF). Crystallographic data of **2b** (CIF). CCDC 1497612. For ESI and crystallographic data in CIF or other electronic format see DOI: 10.1039/c6sc04842k


**DOI:** 10.1039/c6sc04842k

**Published:** 2016-12-21

**Authors:** Meng Gao, Huifang Su, Yuhan Lin, Xia Ling, Shiwu Li, Anjun Qin, Ben Zhong Tang

**Affiliations:** a Guangdong Innovative Research Team , State Key Laboratory of Luminescent Materials & Devices , South China University of Technology , Guangzhou 510640 , China . Email: msqinaj@scut.edu.cn; b Department of Chemistry , Hong Kong Branch of Chinese National Engineering Research Center for Tissue Restoration and Reconstruction , The Hong Kong University of Science & Technology , Clear Water Bay , Kowloon , Hong Kong , China . Email: tangbenz@ust.hk

## Abstract

Photoactivatable probes for lipid droplets (LDs)-specific live-cell imaging are powerful tools for investigating their biological functions through precise spatial and temporal control. Ideal photoactivatable probes for LDs imaging require high concentration accumulation of fluorophores in LDs, simple synthetic procedures, and excellent photoactivation efficiency. However, it is difficult to overcome these challenges by conventional fluorophores due to aggregation-caused quenching (ACQ). In this study, a new class of photoactivatable and LDs-specific fluorescent probes was developed based on dihydro-2-azafluorenones, which can easily undergo photooxidative dehydrogenation reaction to afford 2-azafluorenones with aggregation-induced emission (AIE) properties. Dihydro-2-azafluorenones as photoactivatable and LDs-specific probes display significant advantages of excellent photoactivation efficiency and lack of self-quenching in the aggregated state, and are expected to have broad applications in study of biological functions of LDs' through light-controlled spatiotemporal imaging.

## Abstract 2

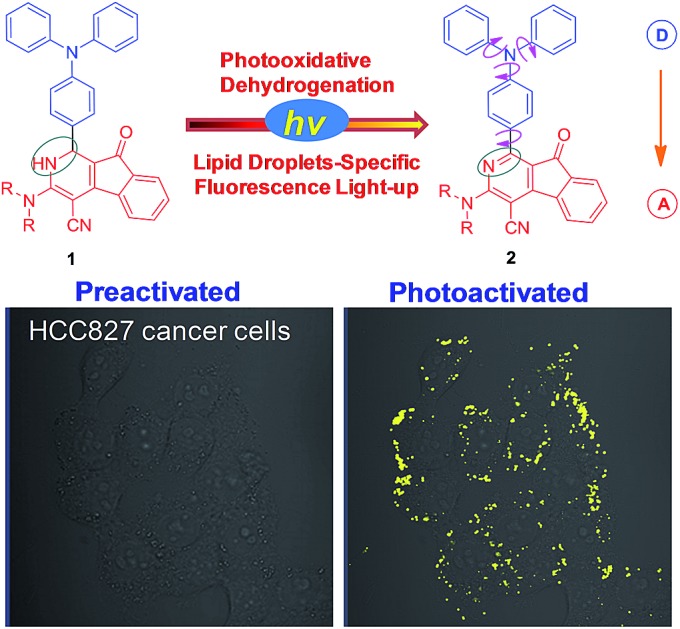
Photoactivatable probes for lipid droplets (LDs)-specific live-cell imaging are powerful tools for investigating their biological functions through precise spatial and temporal control.

## Introduction

Lipid droplets (LDs) as reservoirs of lipids and proteins are dynamic organelles and vary in number, morphology, and size in different cells.^1^ Due to the multifunctions of LDs in energy sources, membrane synthesis, and protein degradation, LDs are linked to various diseases, such as inflammation, virus infection, and obesity.^2^ Recently, the elevated expression level of LDs has been proposed as a biomarker of cancer due to the strong requirement for fatty acids and phospholipids in the rapidly growing cancer cells.^3^ Photoactivatable fluorescent probes are powerful tools for cell biology studies through light-controlled imaging at high spatial and temporal resolution.^4^ It is thus highly desirable to develop LDs-specific photoactivatable probes to investigate their various biological functions.^5^ However, to the best of our knowledge, to date, LDs-specific photoactivatable probes based on small organic molecules, which are less disruptive to the native biology and more convenient for operation than fluorescent proteins, have not been reported in the literature.

To achieve the photoactivatable fluorescent imaging of LDs, two major challenges need to be overcome. First, fluorophores should selectively accumulate in LDs at high concentration to emit bright fluorescence with a high signal-to-noise ratio; however, this is difficult for conventional fluorophores due to aggregation-caused quenching (ACQ).^6^ Second, photoactivatable probes are required to achieve light-controlled spatiotemporal imaging of LDs, but previously reported photoactivatable probes suffer from limited light-up mechanisms, complicated synthetic procedures, generation of toxic byproducts, and difficulty to specifically accumulate in LDs.^7^ Although several LDs-specific fluorescent dyes, such as Nile Red, BODIPY493/503 green, monodansylpentane, AFN, NPBDP, LipidTOX red, and LD540, have been developed, the lack of photoactivatable ability and self-quenching at high concentration have severely limited their applications.^8^

Recently, aggregation-induced emission (AIE) has been proposed as a fundamental solution to solve the fluorescence self-quenching problem in the aggregated state.^9^ The AIE light-up mechanism has been proven to arise from the restriction of intramolecular motion.^10^ AIE-based bioprobes have unique advantages in terms of superior brightness, long-term *in situ* retention ability, high photostability, and low cytotoxicity.^11^ We have recently developed several AIEgens, including TPE-AmAl, TPE-AC, FAS, and DPAS, for LDs-specific imaging with the advantages of absence of self-quenching and easily adjustable emission spectra.^12^ However, it is difficult to introduce photoresponsive groups into these classic AIEgens. Therefore, LDs-specific probes based on a new class of AIEgens with easy availability and excellent photoactivation efficiency are highly required to explore the biological functions of LDs.

**Scheme 1:**
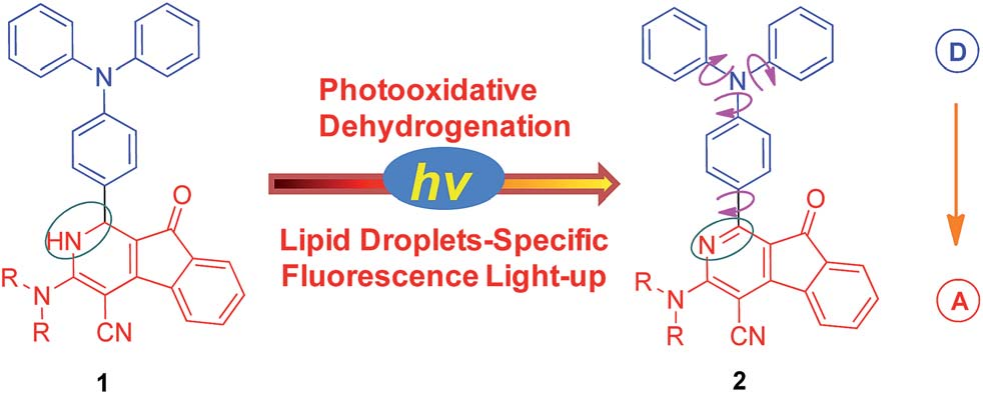
Photooxidative dehydrogenation of dihydro-2-azafluorenone **1** to afford 2-azafluorenone **2** with AIE properties.

2-Azafluorenones are traditionally studied as core structures in many biologically active molecules; however, their photophysical properties and bioimaging applications have been rarely investigated.^13^ Unexpectedly, we found that 2-azafluorenone **2** can emit strong fluorescence in the aggregated state with typical AIE properties *via* the restriction of intramolecular motion and twisted intramolecular charge transfer (TICT) mechanisms (Scheme 1). Moreover, we found that dihydro-2-azafluorenone **1** can be efficiently transformed into 2-azafluorenone **2***via* photooxidative dehydrogenation reaction and can be used for LDs-specific imaging with an excellent photoactivation efficiency.

## Experimental section

### General procedures for the synthesis of compounds **1** and **2** (**1a** and **2a** are presented as an example)

#### Synthesis of **1a**

BDOYM (225 mg, 0.5 mmol) and morpholine (43.5 mg, 0.5 mmol) were first dissolved in MeCN (10 mL). The mixture was stirred at 50 °C under nitrogen for 12 h. After cooling down to room temperature, the reaction mixture was dried under reduced pressure. The residue was further separated by column chromatography (silica, petroleum ether : ethyl acetate = 5 : 1) to give **1a** as a red solid (126 mg, 47% yield).

#### Synthesis of **2a**

BDOYM (225 mg, 0.5 mmol) and morpholine (43.5 mg, 0.5 mmol) were first dissolved in MeCN (10 mL). The mixture was stirred at reflux temperature under dry air and irradiation with a 7 W fluorescent bulb for 12 h. After cooling down to room temperature, the reaction mixture was dried under reduced pressure. The residue was further separated by column chromatography (silica, petroleum ether : ethyl acetate = 8 : 1) to give **1a** as an orange red solid (162 mg, 61% yield).

### Cell cultures

HCC827, A549, and HLF cell lines were purchased from ATCC. HCC827 cells were cultured in RPMI-1640 with 1% penicillin–streptomycin and 10% FBS at 37 °C in a humidified incubator with 5% CO_2_. A549 and HLF cells were cultured in DMEM with 1% penicillin–streptomycin and 10% FBS at 37 °C in a humidified incubator with 5% CO_2_. The culture medium was changed every other day and the cells were collected by treating with 0.25% trypsin–EDTA solution after they reached confluence.

### Cell viability

HCC827, A549, and HLF cells were respectively seeded in 96-well plates at a density of 5 × 10^4^ cells per mL. After 24 h of culture, different concentrations of **1** or **2** were added and further incubated for 24 h. The sample and control wells were washed twice with PBS buffer and added with freshly prepared MTT medium solution (0.5 mg mL^–1^, 100 μL). After 3 h of incubation at 37 °C, the MTT medium solution was carefully removed and washed twice with PBS buffer. DMSO (100 μL) was then added into each well and the plate was gently stirred for 10 min at room temperature to dissolve all the precipitates that were formed. The absorbance of sample and control wells at 570 nm was then measured by a microplate reader. Cell viability was then calculated by the ratio of the absorbance of the sample wells to control cells.

### Cell imaging

Cells were grown in a 35 mm Petri dish with a coverslip at 37 °C. After incubation with **1** (20 μM) for 15 min, the cells were washed with PBS for three times. The photoactivated images were obtained using a confocal microscope *via* increasing scans at 405 nm with 1% laser power (the scanning rate was 22.4 s per frame). For **1a**, the emission filter was 438–604 nm; for **1b**, the emission filter was 515–690 nm; and for **1c**, the emission filter was 551–675 nm.

### Confocal co-localization

For co-staining with lipid dye BODIPY493/503 green, cells were first incubated with probe **1** (20 μM) and BODIPY493/503 green (100 nM) at 37 °C for 15 min. The medium was then removed and the cells were rinsed with PBS for three times, and then imaged using a confocal microscope. For probe **1**, the fluorescence was first photoactivated by irradiation at 405 nm (1% laser power) for designed time intervals, and then the fluorescence images were obtained. For **1a**, the emission filter was 572–607 nm; for **1b**, the emission filter was 572–689 nm; and for **1c**, the emission filter was 572–675 nm; for BODIPY493/503 green, the excitation was 488 nm and the emission filter was 510–553 nm.

### Spatially and temporally controlled cell imaging

The observation window containing multicells was first imaged under irradiation at 405 nm (0.2% power). The selected cells were then irradiated in a bleach model with 405 nm laser (0.2% power) for 20 scans (the scanning rate was 22.4 s per frame). Subsequently, the whole observation window was imaged under irradiation at 405 nm (0.2% power). This process was repeated until all the selected cells in the observation window were lit-up.

## Results and discussion

**Fig. 1:**
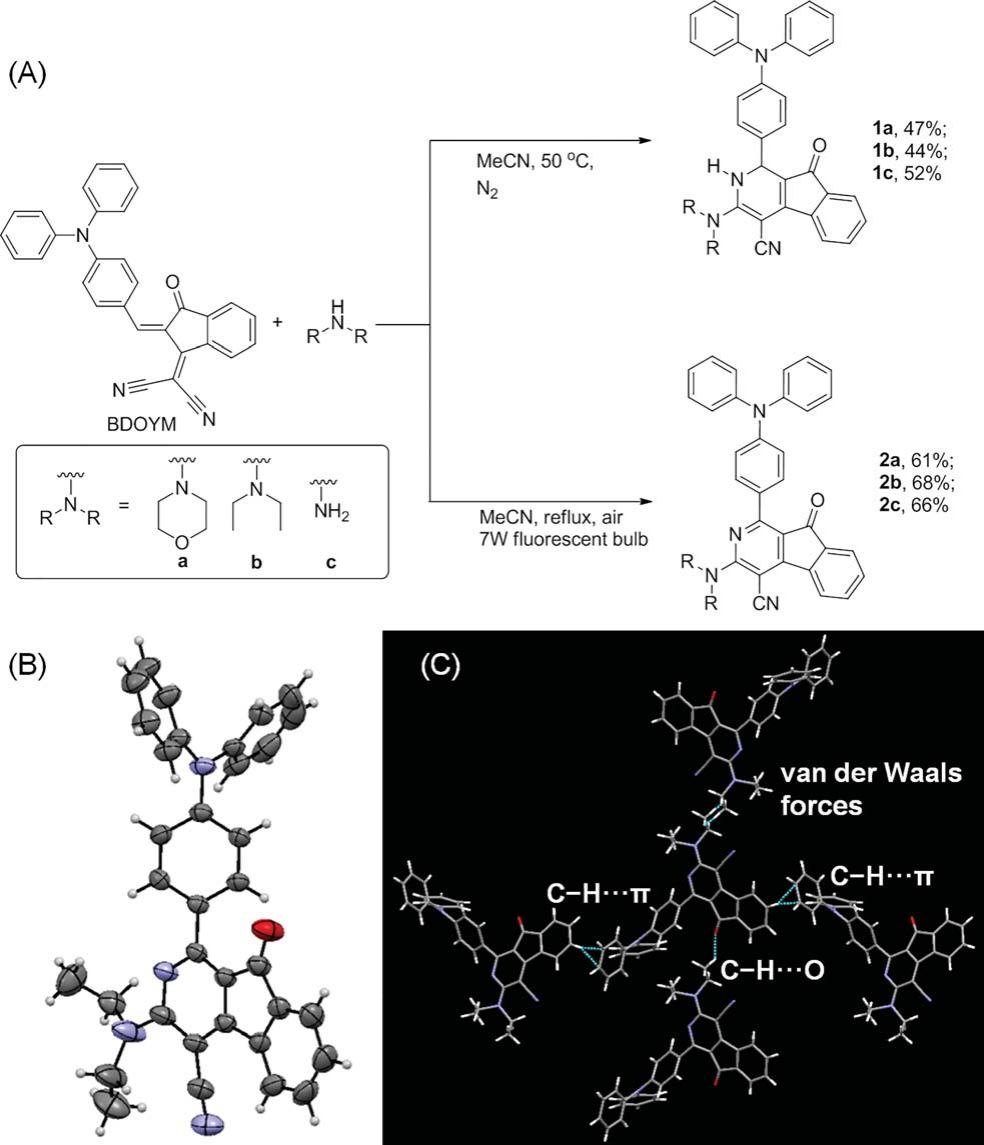
(A) Synthetic routes for dihydro-2-azafluorenone **1** and 2-azafluorenone **2**. (B) Oak ridge thermal ellipsoid plot program (ORTEP) drawing of **2b**. (C) Crystal packing structure of **2b**. Intermolecular C–H···π, C–H···O, and van der Waals interactions are shown in green dashed lines.

We first established an efficient and direct method for preparing dihydro-2-azafluorenone **1** through a one-step reaction from easily available 2-((*Z*)-2-(4-(diphenylamino)benzylidene)-1,2-dihydro-1-oxoinden-3-ylidene)malononitrile (BDOYM) and amines, including morpholine, diethylamine, and ammonia solution (Fig. 1A). We also found that 2-azafluorenone **2** can be directly obtained from BDOYM and amines under light irradiation (7 W fluorescent bulb) and using air as the oxidant (for proposed reaction mechanism, see Scheme S1 in the ESI†). Structures of all obtained compounds were elucidated by NMR spectroscopy and high-resolution mass spectrometry (Fig. S1–S6†). The structure of compound **2b** was also verified by single crystal X-ray diffraction (Fig. 1B and C).^14^ In the crystal of **2b**, the dihedral angle between the planar 2-azafluorenone ring and the 1-linked phenyl ring was 41.9(6)°, whereas the triphenylamine moiety exhibited a propeller conformation with dihedral angles of 61.3(2)°, 67.9(1)°, and 73.3(6)° between the aryl rings, respectively. This kind of conformation can contribute to the non-irradiative release of energy in solution *via* intramolecular rotation. In the crystal packing structure, various intermolecular C–H···π, C–H···O, and van der Waals interactions were observed, which can help to restrict the intramolecular motion and block the non-radiative release of energy in the aggregated state.

**Fig. 2:**
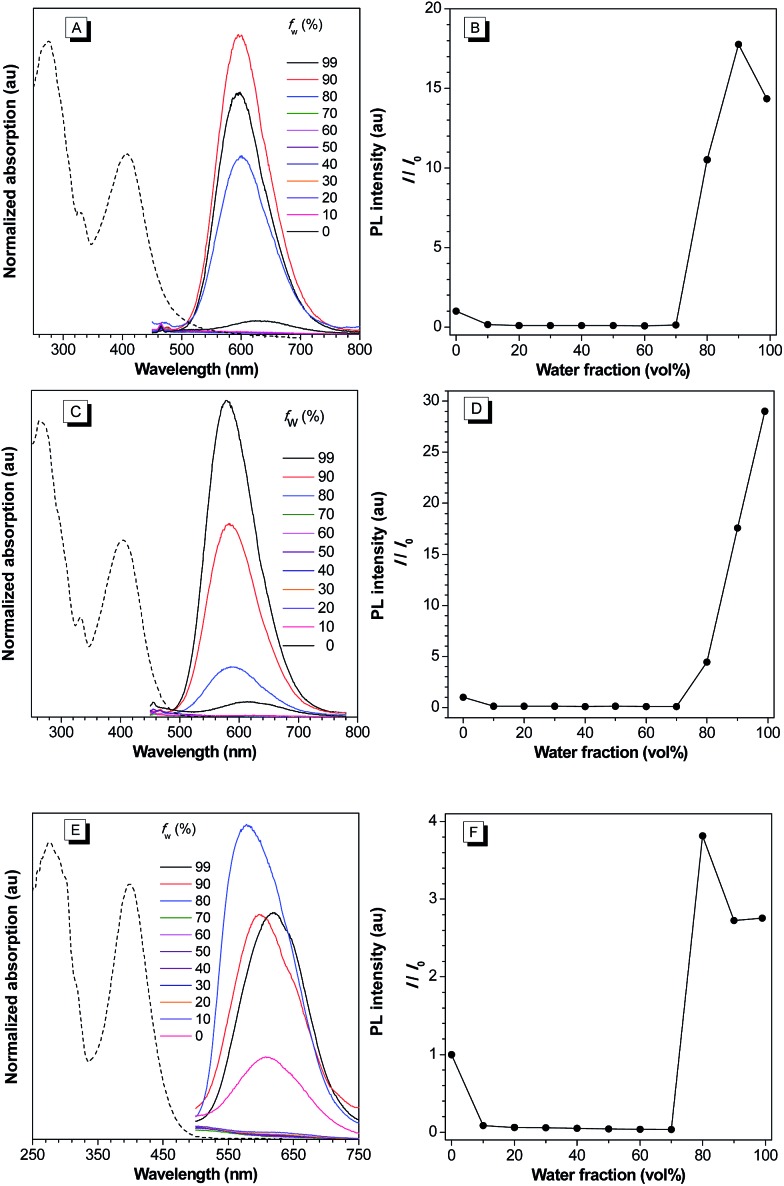
(A, C, and E) Normalized UV-vis absorption spectra of **2a**, **2b**, and **2c** THF (dashed line); PL spectra of **2a**, **2b**, and **2c** in THF and THF/water mixture with different water fractions (*f*_w_). (B, D, and F) Plots of relative maximum emission intensity (*I*/*I*_0_) of **2a**, **2b**, and **2c***versus* the solvent composition of THF/water mixture. [**2a**] = [**2b**] = [**2c**] = 10 μM. For **2a**, *λ*_ex_ = 409 nm. For **2b**, *λ*_ex_ = 402 nm. For **2c**, *λ*_ex_ = 400 nm.

Intrigued by the increased fluorescence intensity of **2** during drying on thin-layer chromatography (TLC) plates, we first examined their photophysical properties. For compound **2a**, an absorption peak at 409 nm and a weak emission peak at 624 nm were observed in dilute THF solution (Fig. 2). In the THF/water mixture, its emission first underwent quenching from water fraction (*f*_w_) = 10 to 70%, and this phenomenon probably originated from the twisted internal charge transfer (TICT) effect,^15^ which was verified by the red-shifted and decreased emission of **2a** with the increasing solvent polarity (Fig. S7†). Density functional theory based calculation of **2a** also verified its typical donor–acceptor structural feature (Fig. S8†). The highest occupied molecular orbital (HOMO) of **2a** is localized on the triphenylamine moiety, whereas the lowest unoccupied molecular orbital (LUMO) is localized on the 2-azafluorenone moiety. Interestingly, further increase in the water fractions (*f*_w_) from 70 to 90% led to a significant emission enhancement, which can be due to the formation of aggregates of **2a** caused by the poor solvating ability of THF/water mixture with high water content. Successively, the emission intensity of **2a** showed a little decrease at 99% water fraction, which can be possibly due to the fast decrease in the solubility leading to the formation of amorphous aggregates with lower fluorescence efficiency.^16^ To verify that the AIE phenomenon is caused by the restriction of intramolecular motion, we measured the emission of **2a** in polar solvents of ethanol and glycerol with low (*η* = 1.2 mPa S) and high viscosity (*η* = 945 mPa S), respectively.^17^ A 158-fold emission enhancement at 600 nm was observed in glycerol than that in ethanol, which clearly verified that the AIE phenomenon is caused by the restriction of intramolecular motion (Fig. S9†).^18^ Similar to **2a**, both **2b** and **2c** first showed a decrease in the fluorescence intensity with the increase in the water fraction (*f*_w_) from 0 to 70%, and then exhibited significant emission enhancements as *f*_w_ increased from 70 to 99% and 80%, respectively. We then measured the photophysical properties of **2a–c** in the film state (Table S1†). Compared to those in the THF solution state, **2a**, **2b**, and **2c** in the film state, respectively, exhibited 20, 18.4, and 12-fold increase in the fluorescence quantum yields (*Φ*_f_), 3.4, 8.8, and 7.0-fold increase in the fluorescence lifetime (*τ*), 2.5, 2.1, and 1.7-fold increase in the radiative decay rates (*k*_r_), and 3.6, 9.2, and 7.6-fold decrease in the nonradiative decay rates (*k*_nr_). They also exhibited large Stokes shifts of 8424, 8668, and 8607 cm^–1^ in the film state, respectively. The excellent emission efficiency enhancement and large Stokes shifts in the aggregated state can greatly favor their applications in bioimaging.

**Fig. 3:**
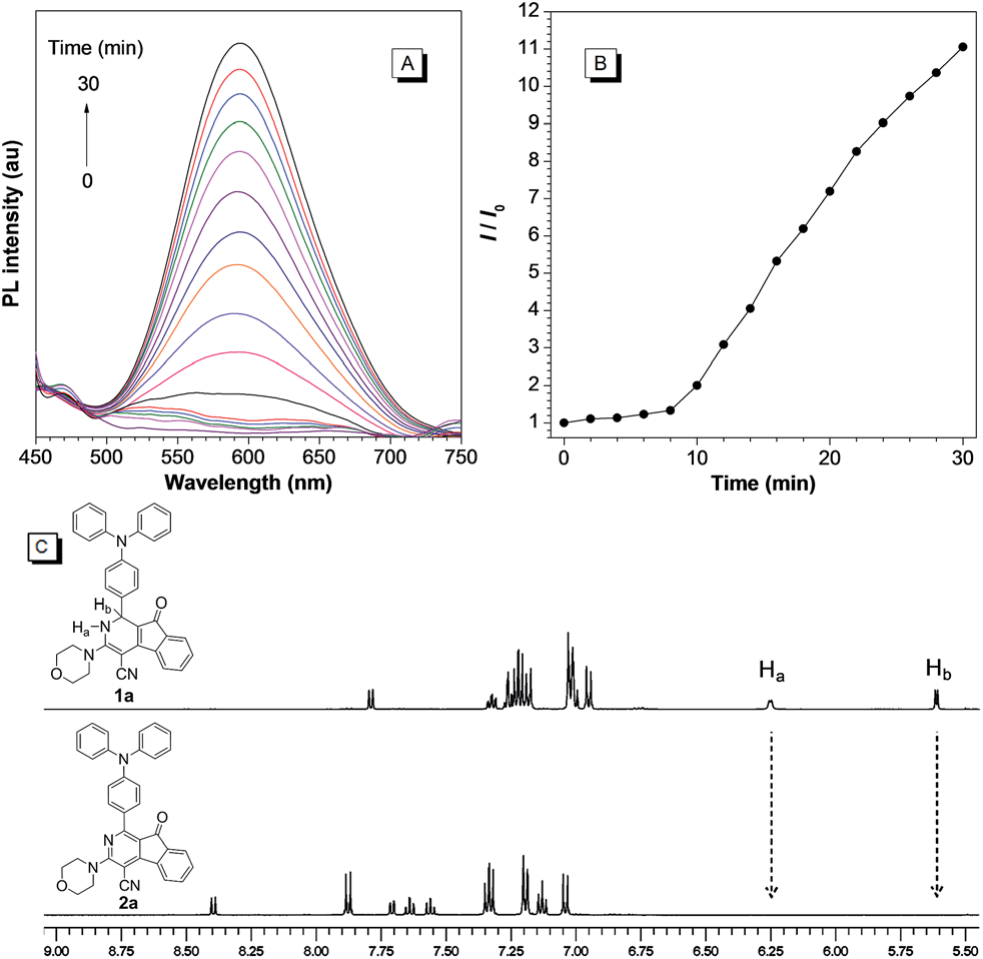
(A) The PL spectra of **1a** in DMSO/water mixture with 99% water fraction under irradiation at 365 nm for 0–30 min. *λ*_ex_ = 409 nm. (B) Plot of relative PL intensity (*I*/*I*_0_) of **1a** at 600 nm *versus* the irradiation time. (C) The stacking ^1^H NMR spectra of **1a** and **2a**.

We next examined the photooxidative transformation of dihydro-2-azafluorenone **1** into 2-azafluorenone **2**. Upon irradiation at 365 nm using a hand-held UV lamp, a gradually increased fluorescence was observed for **1a** in aqueous solution (Fig. 3A and B), indicating that **1a** can efficiently undergo photooxidative transformation to afford AIE-active **2a**. Various water soluble oxidants, such as H_2_O_2_, *t*-BuOOH, NaOCl, sodium persulfate, and potassium peroxymonosulfate, have also been tested for the oxidation of **1a** into **2a** under dark conditions at room temperature; however, all of them can not promote this transformation (Fig. S10†). The photooxidative transformation was also verified by the UV-vis absorption spectra changes of **1a**, in which the maximum absorption wavelength progressively shifted from 530 nm to a shorter wavelength at 413 nm (Fig. S11†). Moreover, the stacking ^1^H NMR spectra of **1a** and **2a** showed that the H_a_ and H_b_ in **1a** (peaks at 6.25 and 5.61 ppm, respectively) completely disappeared in **2a** (Fig. 3C), which clearly verified the proposed photooxidative dehydrogenation mechanism. Similar to **1a**, compounds **1b** and **1c** also exhibited the fast light-up fluorescence and blue-shifted absorption under UV light irradiation (Fig. S12–S15†), which indicates their smooth transformation to **2b** and **2c***via* the photooxidative reaction.

**Fig. 4:**
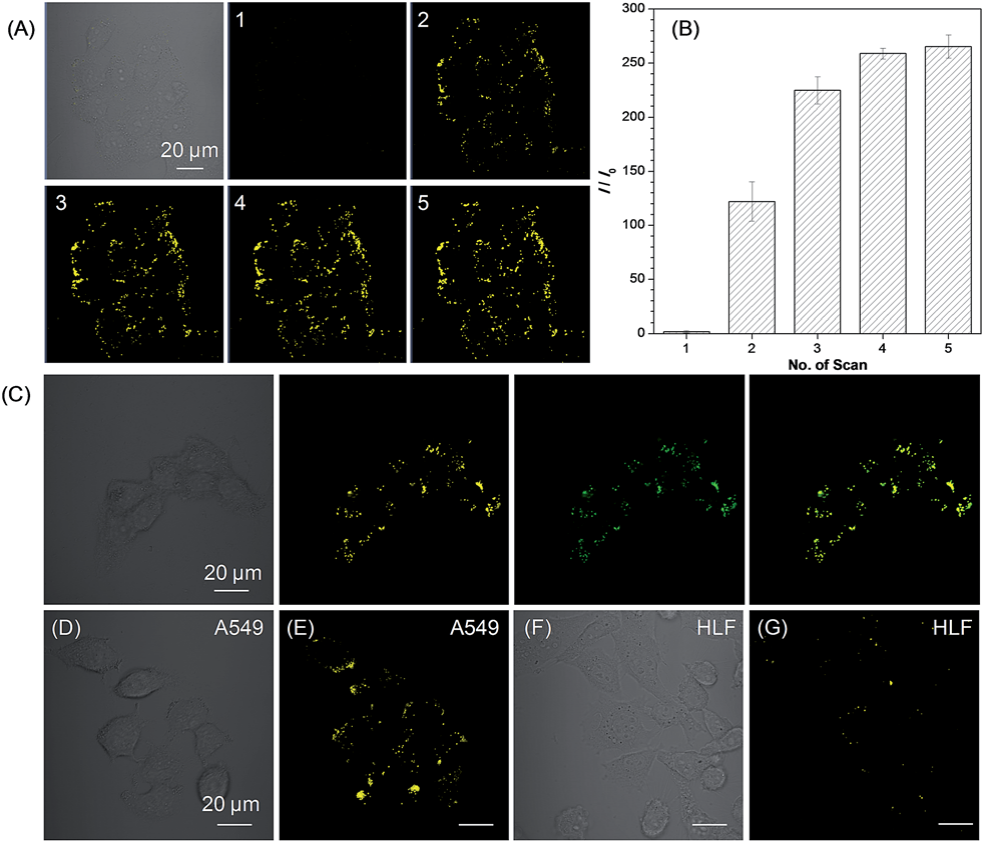
(A) Bright field and fluorescence images of live HCC827 cells obtained under white light illumination and increasing scans at 405 nm with 1% laser power (the number of scans are shown in the upper left corner and the scanning rate was 22.4 s per frame). (B) Plot of fluorescence enhancement (*I*/*I*_0_) of HCC827 cells with the increasing scans at 405 nm. (C) Bright field and fluorescence images of the HCC827 cells stained with **1a** after photoactivation (yellow), BODIPY493/503 (green), and the merged image. (D) Bright field, and (E) fluorescence images of the live A549 cells stained with **1a** after photoactivation. (F) Bright field, and (G) fluorescence images of live HLF cells stained with **1a** after photoactivation by irradiation. [**1a**] = 20 μM. Scale bar = 20 μm.

We then used **1a** as a photoactivatable probe for live-cell imaging. Based on MTT assay, the cytotoxicity of **1a** and **2a** was first evaluated for lung cancer HCC827, A549 cells, and normal lung HLF cells. As shown in Fig. S16,† no significant change in the cell viability was observed even when a high concentration of 100 μM of **1a** or **2a** was present in the culture medium, which suggests that **1a** or **2a** has very low cytotoxicity. Based on **1a**, we then conducted the photoactivatable imaging experiment in the lung cancer HCC827 cells. After incubation at 37 °C for 15 min, **1a** can be efficiently uptaken by HCC827 cells and a very fast light-up process (less than 2 min) was observed *via* irradiation at 405 nm with very mild irradiation of only 1% laser power (Fig. 4A). Through statistically analyzing the increased fluorescence intensity of **1a** in HCC827 cells, an excellent light-up ratio of 265-fold was obtained (Fig. 4B). The co-localization experiment with lipid dye BODIPY493/503 green was then conducted to test the specificity of **1a** for LDs (Fig. 4C). The results show that photoactivated **1a** could co-localize well with BODIPY493/503 green with a high overlap coefficient of 0.99 (Fig. S17†). Through light irradiation at 405 nm, a fast light-up process of **1a** was also observed in the A549 cells (Fig. 4D, E and S18†), and its specificity for LDs was also verified by co-staining with BODIPY493/503 green (Fig. S19†). Compared to lung cancer HCC827 and A549 cells, only few LDs were observed in the normal lung HLF cells by photoactivated **1a** (Fig. 4F and G). The much larger number of LDs in the lung cancer HCC827 and A549 cells is probably due to their faster growing rate compared with normal lung HLF cells.^3^ This experiment suggests that **1a** as a photoactivatable and LDs-specific probe can be used to discriminate between the cancer and normal cells through their different expression levels for LDs.

**Fig. 5:**
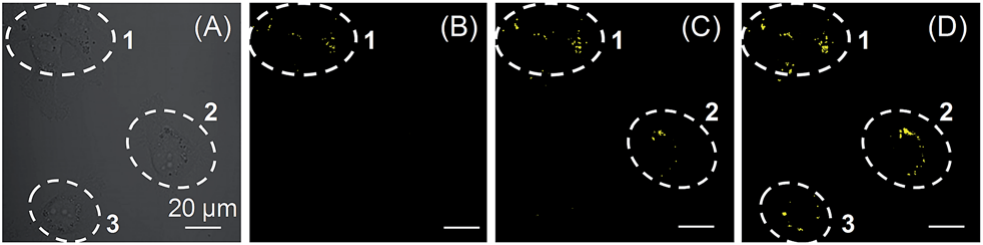
(A) Bright field of HCC827 cells. (B–D) Sequential photoactivation of HCC827 cells (cells 1, 2, and 3) by irradiation at 405 nm (0.2% laser power). Dashed lines indicate the periphery of cells. [**1a**] = 20 μM. Scale bar = 20 μm.

Light as an external trigger is a valuable and easily controllable tool with high spatial and temporal accuracy. As shown in Fig. 5, sequential photoactivation of **1a** in selected HCC827 cells can be achieved in a multicellular environment. This experiment suggests that **1a** as a photoactivatable probe is potential to monitor the dynamic events of LDs in a complex biological sample.

**Fig. 6:**
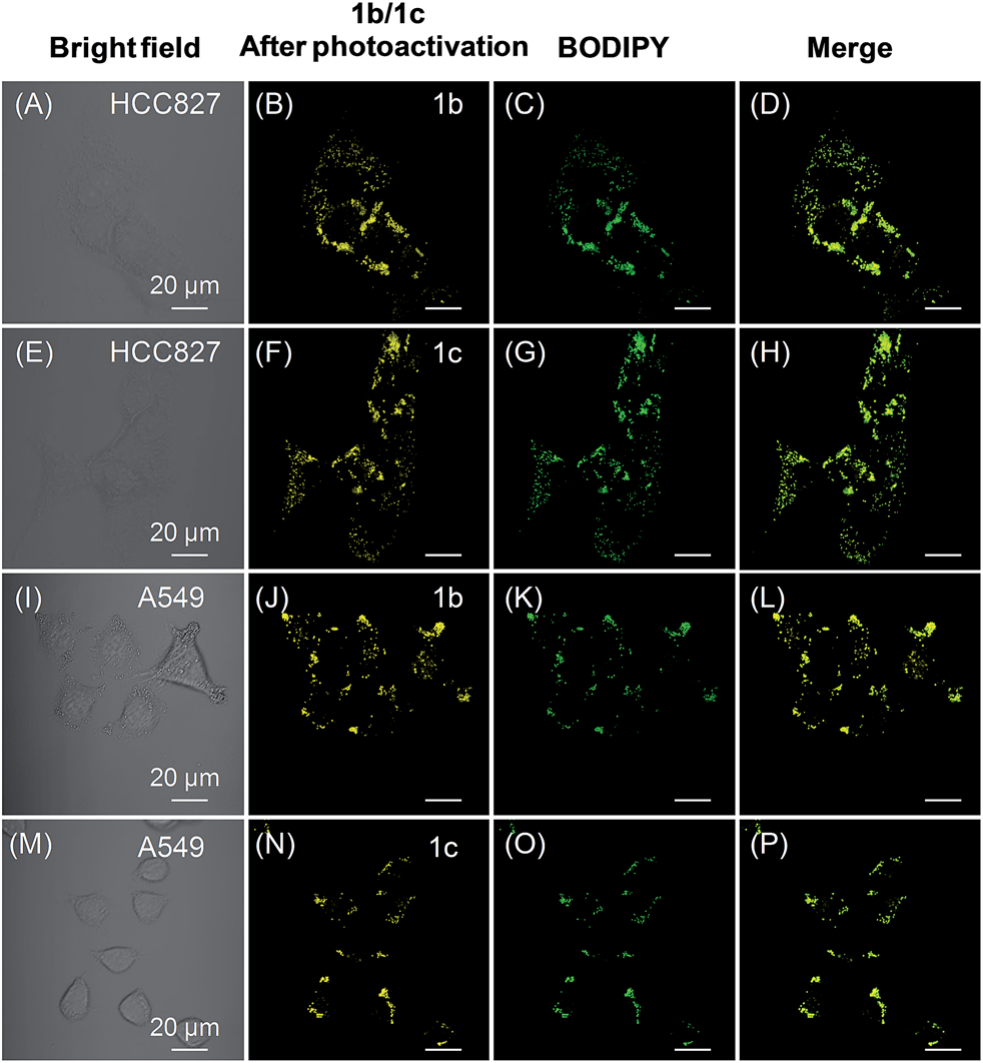
(A and E) Bright-field images of HCC827 cells. (B and F) Fluorescence images of HCC827 cells from **1b** and **1c** after photoactivation, respectively. (C and G) Fluorescence images of HCC827 cells from BODIPY493/503 green. (D and H) The merged images. (I and M) Bright-field images of A549 cells. (J and N) Fluorescence images of A549 cells from **1b** and **1c** after photoactivation, respectively. (K and O) Fluorescence images of A549 cells from BODIPY493/503 green. (L and P) The merged images. Both **1b** and **1c** were photoactivated through irradiation at 405 nm for 3 min (1% laser power). [**1b**] = [**1c**] = 20 μM. Scale bar = 20 μm.

We also tested the targeting specificity and photoactivatable imaging ability of **1b–c** for LDs in the lung cancer HCC827 and A549 cells. Through irradiation at 405 nm with 1% laser power, fast light-up processes were observed for both **1b** and **1c** in HCC827 and A549 cells, and maximum emission intensities could be achieved in 3 min (Fig. S20–S23†). The co-localization experiment with lipid dye BODIPY493/503 green clearly showed that **1b** and **1c** can also be used for LDs-specific photoactivatable imaging (Fig. 6). In HCC827 cells, the overlap coefficients for **1b** and **1c** with lipid dye BODIPY493/503 green are 0.99 and 0.98, respectively; whereas in A549 cells, the overlap coefficients for **1b** and **1c** with BODIPY493/503 green are 0.97 and 0.99, respectively.

## Conclusions

In conclusion, we have developed photoactivatable and LDs-specific probes based on dihydro-2-azafluorenones, which can easily undergo photooxidative dehydrogenation reaction to afford 2-azafluorenones with typical AIE properties. Even with different amine substituents, dihydro-2-azafluorenones are generally applicable for LDs-specific imaging in live cells with an excellent photoactivation efficiency and high signal-to-noise ratio. They can also be used for the sequential photoactivation of selected cells in a multicellular environment. Moreover, they can efficiently discriminate between lung cancer and normal lung cells through their different expression levels for LDs. Benefiting from the advantages of absence of self-quenching in the aggregated state, easy preparation, fast cell uptake, low cytotoxicity, and excellent photoactivation efficiency, the photoactivatable AIE probes developed in this study are expected to have broad applications in the biological studies of LDs.

## Supplementary Material

Supplementary information

Crystal structure data

## References

[cit1] Olofsson S. O., Bostrom P., Andersson L., Rutberg M., Perman J., Boren J. (2009). Biochim. Biophys. Acta, Mol. Cell Biol. Lipids.

[cit2] Miyanari Y., Atsuzawa K., Usuda N., Watashi K., Hishiki T., Zayas M., Bartenschlager R., Wakita T., Hijikata M., Shimotohno K. (2007). Nat. Cell Biol..

[cit3] Abramczyk H., Surmacki J., Kopec M., Olejnik A. K., Lubecka-Pietruszewska K., Fabianowska-Majewska K. (2015). Analyst.

[cit4] Raymo F. M. (2013). Phys. Chem. Chem. Phys..

[cit5] Kory N., Thiam A. R., Farese Jr R. V., Walther T. C. (2015). Dev. Cell.

[cit6] MacDonald R. I. (1990). J. Biol. Chem..

[cit7] Zhang Y., Swaminathan S., Tang S., Garcia-Amoros J., Boulina M., Captain B., Baker J. D., Raymo F. M. (2015). J. Am. Chem. Soc..

[cit8] Daemen S., van Zandvoort M. A. M. J., Parekh S. H., Hesselink M. K. C. (2016). Molecular Metabolism.

[cit9] Lim X. (2016). Nature.

[cit10] Mei J., Hong Y., Lam J. W., Qin A., Tang Y., Tang B. Z. (2014). Adv. Mater..

[cit11] Ding D., Li K., Liu B., Tang B. Z. (2013). Acc. Chem. Res..

[cit12] Wang Z., Gui C., Zhao E., Wang J., Li X., Qin A., Zhao Z., Yu Z., Tang B. Z. (2016). ACS Appl. Mater. Interfaces.

[cit13] (a) Heintzelman G. R., Bullington J. L. and Rupert K. C., WO2005042500A1, 2005.

[cit14] CCDC 1497612 contains the supplementary crystallographic data for this paper

[cit15] Hu R. R., Lager E., Aguilar-Aguilar A., Liu J. Z., Lam J. W. Y., Sung H. H. Y., Williams I. D., Zhong Y. C., Wong K. S., Pena-Cabrera E., Tang B. Z. (2009). J. Phys. Chem. C.

[cit16] Shao A., Xie Y., Zhu S., Guo Z., Zhu S., Guo J., Shi P., James T. D., Tian H., Zhu W. H. (2015). Angew. Chem., Int. Ed..

[cit17] Haidekker M. A., Brady T. P., Lichlyter D., Theodorakis E. A. (2005). Bioorg. Chem..

[cit18] Hong Y., Lam J. W. Y., Tang B. Z. (2009). Chem. Commun..

