# Supplementary material for: Photoactivatable aggregation-induced emission probes for lipid droplets-specific live cell imaging
Source: Chem Sci. 2016 Dec 21;8(3):1763–8. doi: 10.1039/c6sc04842k (PMC5933432; doi:10.1039/c6sc04842k)
Supplement: Supplementary file 1 [file SC-008-C6SC04842K-s001.pdf]

Electronic Supplementary Material (ESI) for Chemical Science.
This journal is © The Royal Society of Chemistry 2016

Supporting Information

Photoactivatable aggregation-induced emission probes for lipid droplets-specific live cell imaging

Meng Gao,[ǂa] Huifang Su,[ǂb] Yuhan Lin,[a] Xia Ling,[a] Shiwu Li,[a] Anjun Qin,*[a] Ben Zhong Tang*[a,b]

[a]Guangdong Innovative Research Team, State Key Laboratory of Luminescent Materials & Devices, South China University of Technology, Guangzhou 510640, China

[b]Department of Chemistry and Hong Kong Branch of Chinese National Engineering Research Center for Tissue Restoration and Reconstruction, The Hong Kong University of Science & Technology, Clear Water Bay, Kowloon, Hong Kong, China.

*Corresponding Author: msqinaj@scut.edu.cn; tangbenz@ust.hk

Table of Contents

Materials and Chemicals	S3
Equipment and Methods	S3
NMR and HRMS data of compounds **1** and **2**	S4
**Table S1** Photophysical properties of compounds **2**	S8
**Scheme S1.** Proposed reaction mechanism	S8
**Fig. S1.** ^{1}H and ^{13}C NMR spectra of **1a**	S9
**Fig. S2.** ^{1}H and ^{13}C NMR spectra of **1b**	S10
**Fig. S3.** ^{1}H and ^{13}C NMR spectra of **1c**	S11
**Fig. S4.** ^{1}H and ^{13}C NMR spectra of **2a**	S12
**Fig. S5.** ^{1}H and ^{13}C NMR spectra of **2b**	S13
**Fig. S6.** ^{1}H and ^{13}C NMR spectra of **2c**	S14
**Fig. S7.** Solvent-dependent UV-Vis and PL spectra of **2a**	S15
**Fig. S8.** Molecular orbital amplitude plots of HOMOs and LUMOs of **2**	S15
**Fig. S9.** PL spectra of **2a** in ethanol and glycerol	S15
**Fig. S10.** Fluorescence responses of **1a** with various oxidants	S16
**Fig. S11.** UV-Vis spectra changes of **1a** under UV light irradiation	S16
**Fig. S12.** PL spectra changes of **1b** under UV light irradiation	S17
**Fig. S13.** UV-Vis spectra changes of **1b** under UV light irradiation	S17
**Fig. S14.** PL spectra changes of **1c** under UV light irradiation	S18
**Fig. S15.** UV-Vis spectra of **1c** under UV light irradiation	S18
**Fig. S16.** Cell viabilities of HCC827 cells, A549 cells, and HLF cells	S19
**Fig. S17.** Intensity correlation plots of photoactivated probes **1** and BODIPY493/503 Green	S20
**Fig. S18.** Photoactivatable imaging of A549 cells stained with **1a**	S20
**Fig. S19.** Fluorescent images of A549 cells co-stained with **1a** and BODIPY493/503 Green	S21
**Fig. S20.** Photoactivatable imaging of HCC827 cells stained with **1b**	S21
**Fig. S21.** Photoactivatable imaging of HCC827 cells stained with **1c**	S22
**Fig. S22.** Photoactivatable imaging of A549 cells stained with **1b**	S23
**Fig. S23.** Photoactivatable imaging of A549 cells stained with **1c**	S24

Materials and Chemicals

2-((*Z*)-2-(4-diphenyl-amino)-benzylidene)-1,2-dihydro-1-oxoinden-3-ylidene)-malononitrile (BDOYM) was prepared according to the literature.[1] 1,3-Indanedione was purchased from Acros; malononitrile was purchased from Alfa-Aesar; 4-(*N*, *N*-diphenylamino)benzaldehyde and morpholine were purchased from Energy Chemical; diethylamine was purchased from Aladdin; ammonia solution (25–28 wt.%) was purchased from Guangzhou Chemical Reagent Factory; other chemicals were purchased from Sigma-Aldrich. THF was distilled from sodium under dry nitrogen prior to use.

Dulbecco's Modified Essential Medium (DMEM) and RPMI-1640 were purchased from Gibco (Life Technologies). Ultra pure water was supplied by Milli-Q Plus System (Millipore Corporation, United States). Phosphate buffered saline (PBS), fetal bovine serum (FBS), penicillin, streptomycin, and BODIPY 493/503 were purchased from Thermo Fisher Scientific.

Equipment and Methods

UV-Vis absorption spectra were measured on a Shimadzu UV-2600 spectrophotometer, medium scanning rate, and quartz cuvettes of 1 cm path length. Photoluminescence spectra were recorded on a Horiba Fluoromax-4 spectrofluorometer. The absolute fluorescence quantum yield was measured using a Hamamatsu quantum yield spectrometer C11347 Quantaurus_QY. The fluorescence lifetime was measured using a Hamamatsu Compact Fluorescence Lifetime Spectrometer C11367. ^{1}H and ^{13}C NMR spectra were measured on a Bruker AV 500 NMR spectrometer. High resolution mass spectra (HRMS) were recorded on a GCT Premier CAB 048 mass spectrometer operated in MALDI-TOF model. Single crystal

of **2b** was grown from hexane/dichloromethane via solute solution diffusion method. Single crystal X-ray diffraction intensity data were collected on a Bruker–Nonices Smart Apex CCD diffractometer with graphite monochromated MoKα radiation. Processing of the intensity data was carried out using the SAINT and SADABS routines, and the structure and refinement were conducted using the SHELTL suite of X-ray programs (version 6.10). Confocal lasing scanning microscopic (CLSM) images were obtained on the confocal microscope (Zeiss Laser Scanning Confocal Microscope; LSM7 DUO). The ground-state geometries of **2** were optimized using the density function theory (DFT) method with B3LYP hybrid functional at the basis set level of 6-31G (d, p). All the calculations were performed using Gaussian 09 package.

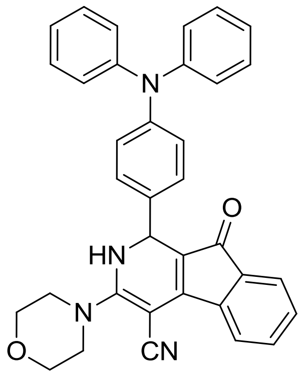

Compound **1a**: Yield 47% (126 mg); red solid; ^{1}H NMR ($CDCl_3$, 500 MHz): δ 7.79 (d, J = 7.5 Hz, 1H), 7.33 (td, J_1 = 7.0 Hz, J_2 = 2.0 Hz, 1H), 7.26–7.18 (m, 8H), 7.03–7.00 (m, 6H), 6.95 (d, J = 8.5 Hz, 2H), 6.25 (d, J = 4.5 Hz, 1H), 5.61 (d, J = 4.5 Hz, 1H), 3.83–3.74 (m, 6H), 3.49–3.46 (m, 2H); ^{13}C NMR ($CDCl_3$, 125 MHz): 190.6, 162.3, 155.8, 149.2, 148.8, 140.8, 136.8, 136.0, 133.1, 130.9, 130.6, 128.5, 125.9, 124.5, 122.1, 121.8, 120.8, 113.5, 67.7, 62.9, 50.6. HRMS (ESI): m/z $[M + Na]^+$ calcd for $C_{35}H_{28}N_4NaO_2$, 559.2110; found, 559.2161.

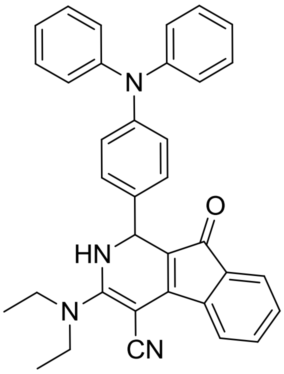

Compound **1b**: Yield 44% (114 mg); red solid; ^{1}H NMR (CD_2Cl_2, 500 MHz): δ 7.89 (d, J = 7.5 Hz, 1H), 7.31 (td, J_1 = 7.0 Hz, J_2 = 2.0 Hz, 1H), 7.27–7.20 (m, 8H), 7.02–6.95 (m, 8H), 6.02 (d, J = 4.0 Hz, 1H), 5.59 (d, J = 4.0 Hz, 1H), 3.60 (q, J = 6.5 Hz, 4H), 1.28 (t, J =6.5 Hz, 6H); ^{13}C NMR (CD_2Cl_2, 125 MHz): 189.9, 156.0, 156.3, 149.0, 148.9, 141.1, 137.5, 136.5, 132.7 130.7, 130.6, 128.4, 125.8, 124.7, 124.4, 122.2, 121.5, 112.6, 61.2, 46.3, 14.7; HRMS (ESI): m/z $[M + Na]^+$ calcd for $C_{35}H_{30}N_4NaO$, 545.2317; found, 545.2379.

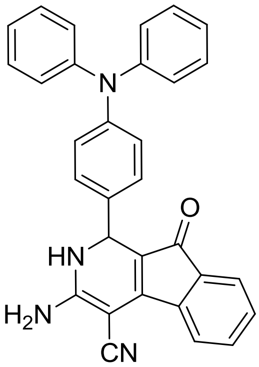

Compound **1c**: Yield 52% (121 mg); red solid; ^{1}H NMR (DMSO-d_6, 500 MHz): 8.19 (d, J = 3.0 Hz, 1H), 7.70 (d, J = 7.0 Hz, 1H), 7.42 (br s, 2H), 7.39 (td, J_1 = 7.5 Hz, J_2 = 1.0 Hz, 1H), 7.32 (td, J_1 = 8.0 Hz, J_2 = 1.0 Hz, 1H), 7.29–7.26 (m, 4H), 7.22–7.21 (m, 3H), 7.02 (tt, J_1 = 7.5 Hz, J_2 = 1.0 Hz, 2H), 6.97–6.95 (m, 6H), 5.51 (d, J = 3.0 Hz, 1H); ^{13}C NMR (DMSO-d_6, 125 MHz): δ 186.3, 158.9, 153.0, 147.1, 146.6, 138.5,

137.9, 135.3, 131.0, 129.8, 129.5, 127.5, 123.7, 122.9, 119.8, 119.7, 118.7, 107.9, 57.8, 52.3; HRMS (ESI): m/z $[M + Na]^+$ calcd for $C_{31}H_{23}N_4NaO$, 489.1691; found, 489.1845.

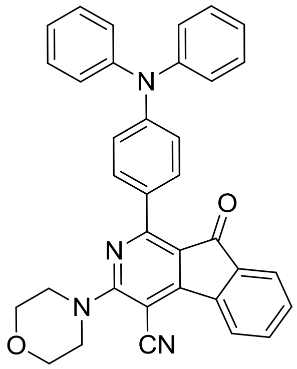

Compound **2a**: Yield 61% (162 mg); orange red solid; ^1H NMR (CD_2Cl_2, 500 MHz): δ 8.39 (d, J = 7.5 Hz, 1H), 7.88 (td, J_1 = 9.0 Hz, J_2 = 2.0 Hz, 2H), 7.71 (dt, J_1 = 7.5 Hz, J_2 = 0.5 Hz, 1H), 7.64 (td, J_1 = 7.5 Hz, J_2 = 1.0 Hz, 1H), 7.56 (dt, J_1 = 7.5 Hz, J_2 = 1.0 Hz, 1H), 7.35–7.32 (m, 4H), 7.20–7.19 (m, 4H), 7.14 (dt, J_1 = 7.5 Hz, J_2 = 1.0 Hz, 1H), 7.12 (dt, J_1 = 7.5 Hz, J_2 = 1.0 Hz, 1H), 7.04 (dt, J_1 = 9.0 Hz, J_2 = 2.5 Hz, 2H), 4.00 (t, J = 5.0 Hz, 4H), 3.85 (t, J = 5.0Hz, 4H); ^{13}C NMR (CD_2Cl_2, 125 MHz): 189.4, 162.2, 161.9, 159.5, 151.7, 148.3, 139.7, 136.8, 135.5, 133.6, 132.8, 130.8, 130.2, 127.0, 125.5, 125.0, 124.5 121.2, 118.5, 117.1, 85.6, 68.0, 49.9. HRMS (ESI): m/z [M + Na]$^+$ calcd for $C_{35}H_{26}N_4NaO_2$, 557.1953; found, 557.1946.

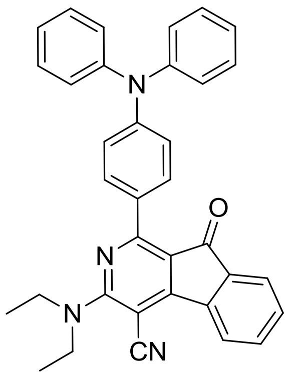

Compound **2b**: Yield 68% (177 mg); orange red solid; ^{1}H NMR ($CDCl_3$, 500 MHz): δ 8.39 (d, J = 8.0 Hz, 1H), 7.89 (d, J = 8.5 Hz, 2H), 7.71 (d, J = 7.0 Hz, 1H), 7.60 (td, J_1 = 7.5 Hz, J_2 = 1.0 Hz, 1H), 7.51 (td, J_1 = 7.5 Hz, J_2 = 1.0 Hz, 1H), 7.32–7.29 (m, 4H), 7.21–7.19 (m, 4H), 7.11–7.06 (m, 4H), 3.90 (q, J = 7.0 Hz, 4H), 1.39 (t, J = 7.0 Hz, 6H); ^{13}C NMR ($CDCl_3$, 125 MHz): 188.1, 161.1, 158.6, 158.3, 150.1, 147.0, 138.4, 135.7, 133.8, 131.9, 131.3, 129.4, 129.3, 125.6, 123.9, 123.5, 123.3, 120.2, 118.1, 114.2, 81.2, 45.0, 13.8. HRMS (ESI): m/z $[M + Na]^+$ calcd for $C_{35}H_{28}N_4NaO$, 543.2161; found, 543.2218.

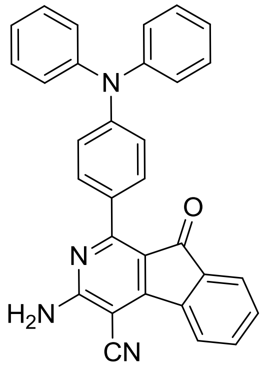

Compound **2c**: Yield 66% (153 mg); orange red solid; ^{1}H NMR ($CDCl_3$, 500 MHz): 8.18 (d, J = 8.0 Hz, 1H), 8.03 (br s, 2H), 7.78–7.77 (m, 3H), 7.66–7.65 (m, 2H), 7.38 (t, J = 8.0 Hz, 4H), 7.16–7.12 (m, 6H), 6.95 (d, J = 8.0 Hz, 2H); ^{13}C NMR (DMSO-d_6, 125 MHz): δ 187.3, 161.6, 160.4, 158.4, 149.8, 147.0, 137.8, 135.6, 134.8, 133.1, 131.8, 130.2, 129.6, 125.5, 124.5, 124.0, 122.7, 120.2, 115.9, 113.8, 81.9; HRMS (ESI): m/z $[M + H]^+$ calcd for $C_{31}H_{21}N_4O$, 465.1715; found, 465.1785.

Table S1. Photophysical properties of 2-azafluorenones **2** in THF solution (Soln) and solid (Solid) states.

**2**	Soln[a)]						Solid[b)]					
	λ_{ab} [nm][c)]	λ_{em} [nm][d)]	Φ_f [%][e)]	τ (ns)[f)]	k_r [10^6 S^{-1}][g)]	k_{nr} [10^8 S^{-1}][h)]	λ_{ab} [nm][c)]	λ_{em} [nm][d)]	Φ_f [%][e)]	τ (ns)[f)]	k_r [10^6 S^{-1}][g)]	k_{nr} [10^8 S^{-1}][h)]
**2a**	409	624	0.6	1.16	5.17	8.57	420	620	12.0	3.95	12.7	2.41
**2b**	402	617	0.5	0.66	7.58	15.1	411	571	9.2	5.80	15.9	1.57
**2c**	400	610	0.8	1.09	7.34	9.10	414	609	9.6	7.58	12.7	1.19

a) In THF solution with a concentration of 10^{-5} M; b) Thin solid film; c) Maximum absorption wavelength; d) Maximum emission wavelength; e) Absolute quantum yield; f) Average fluorescence lifetime; g) Radiative relaxation rate $k_r = \Phi/\tau$; h) Non-radiative relaxation rate $k_{nr} = (1-\Phi)/\tau$.

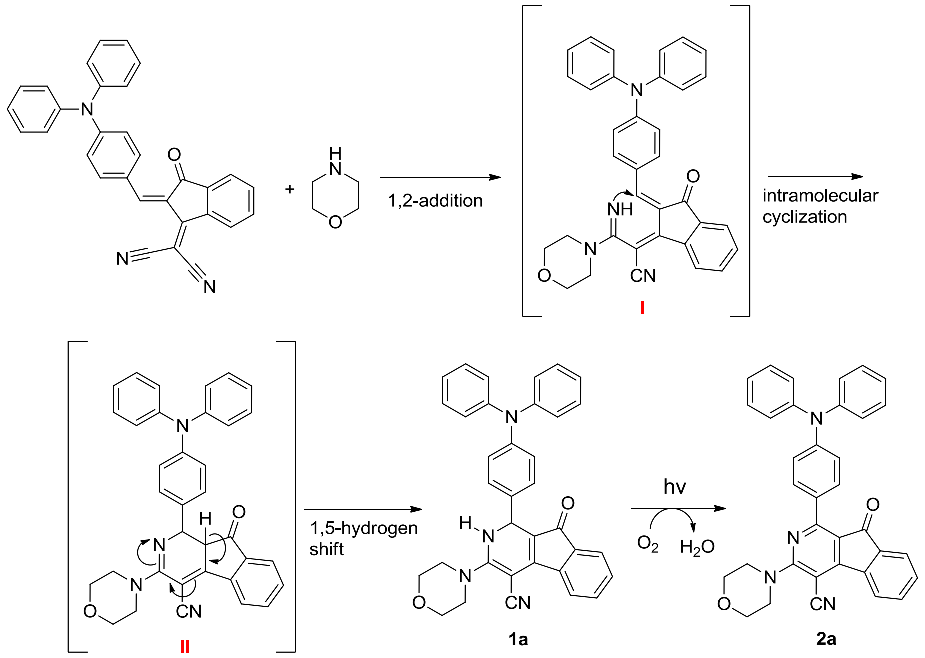

Scheme S1. Proposed reaction mechanism.

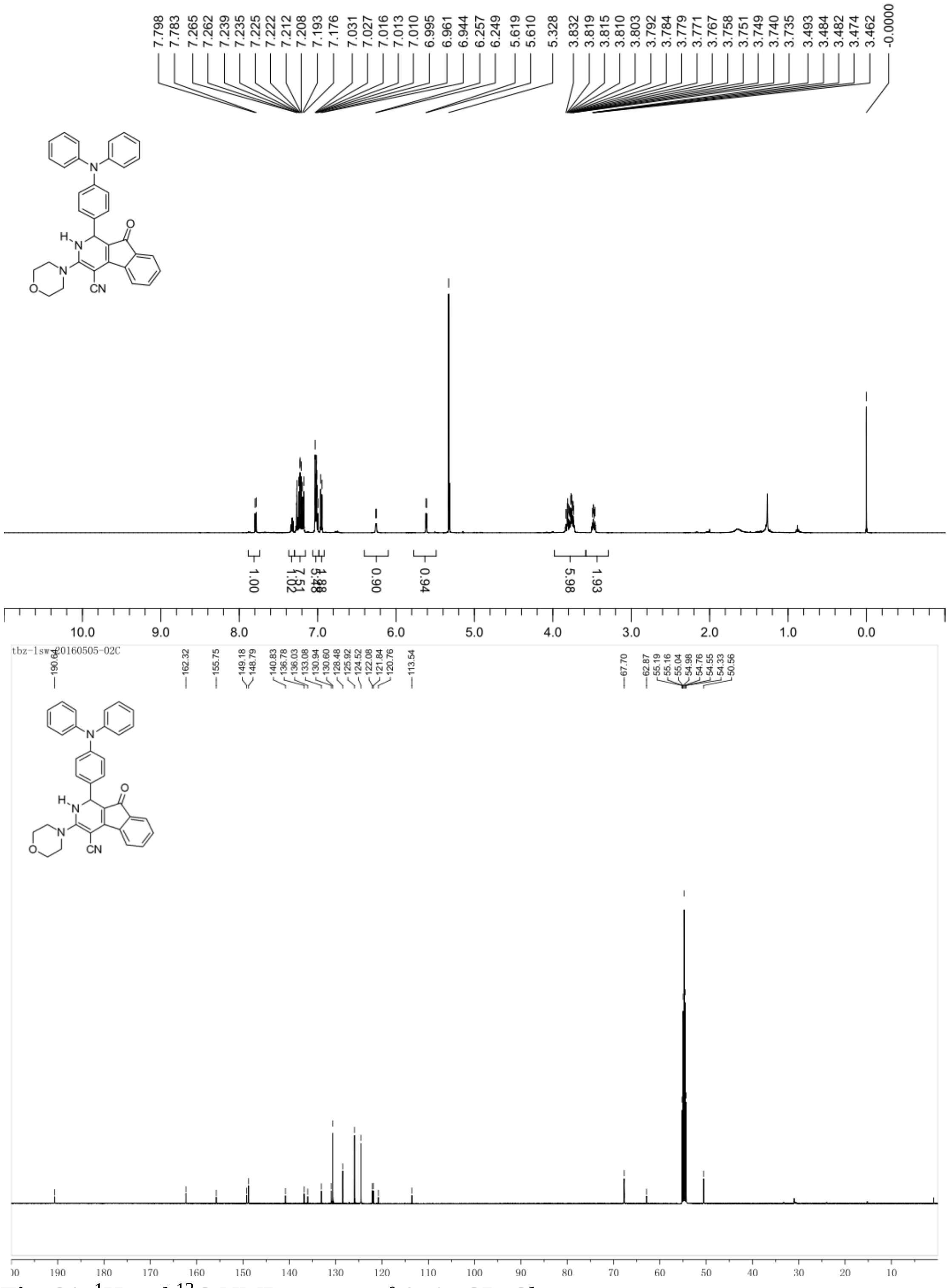

Fig. S1. ^{1}H and ^{13}C NMR spectra of **1a** in CD_2Cl_2.

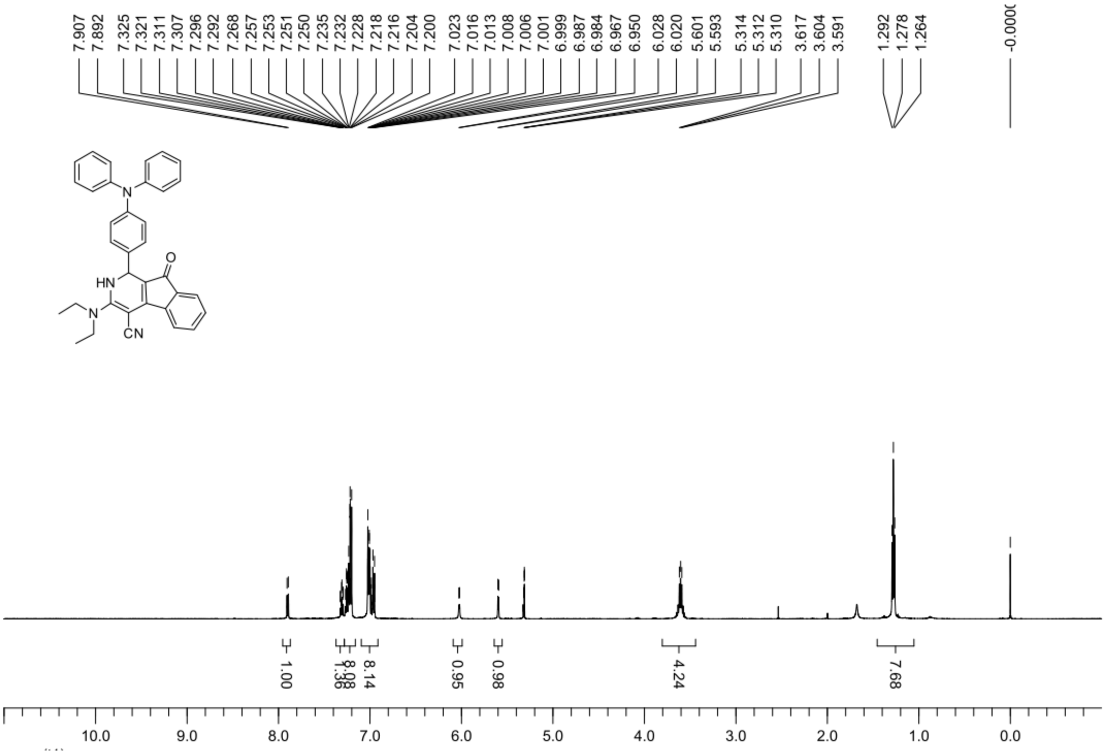

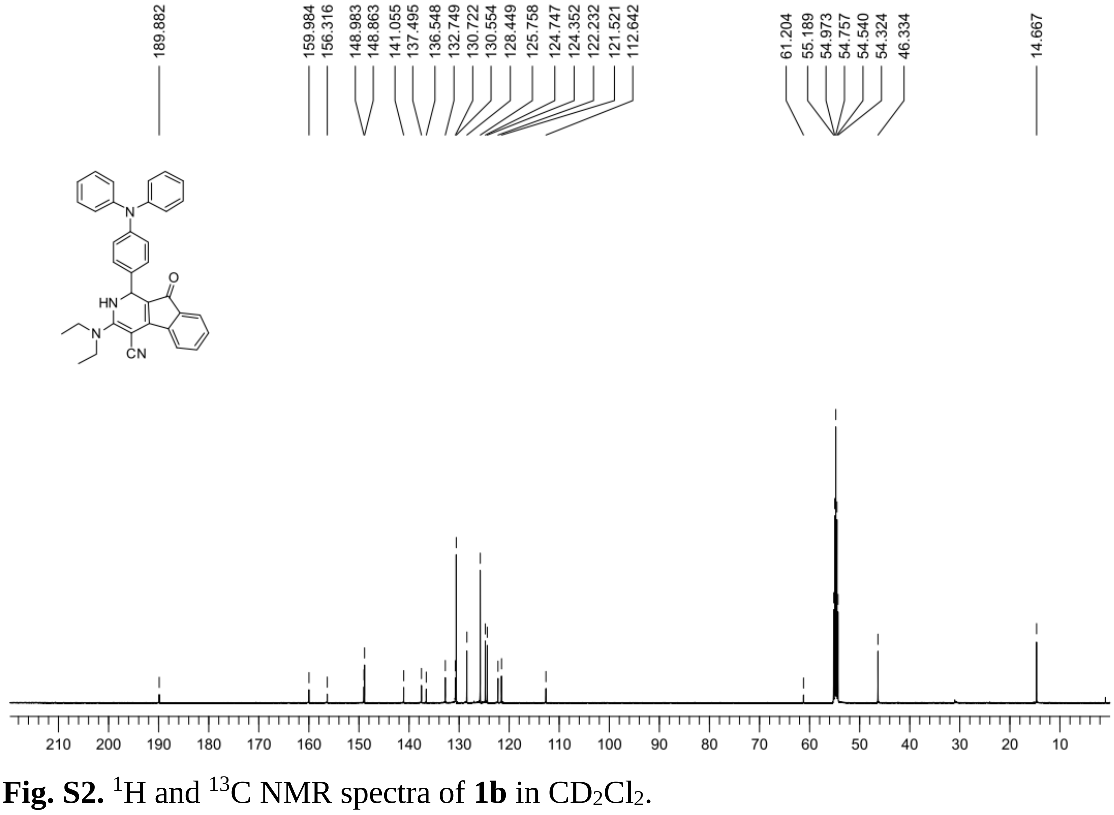

Fig. S2. ^{1}H and ^{13}C NMR spectra of **1b** in CD_2Cl_2.

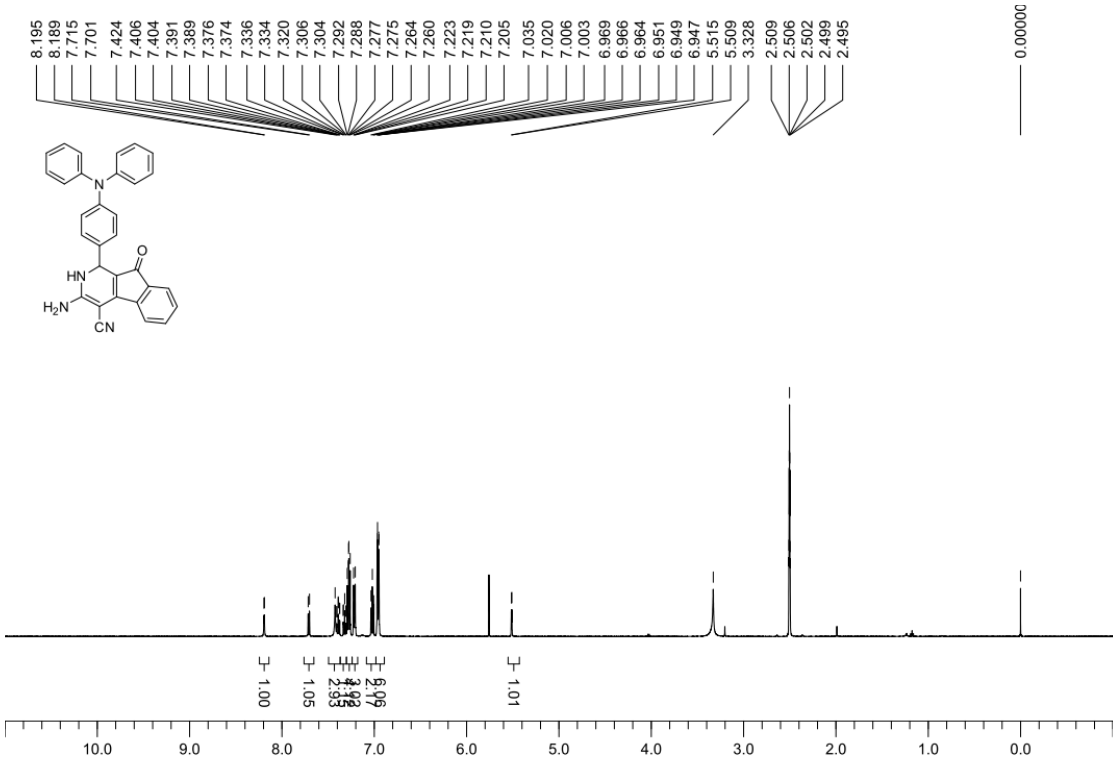

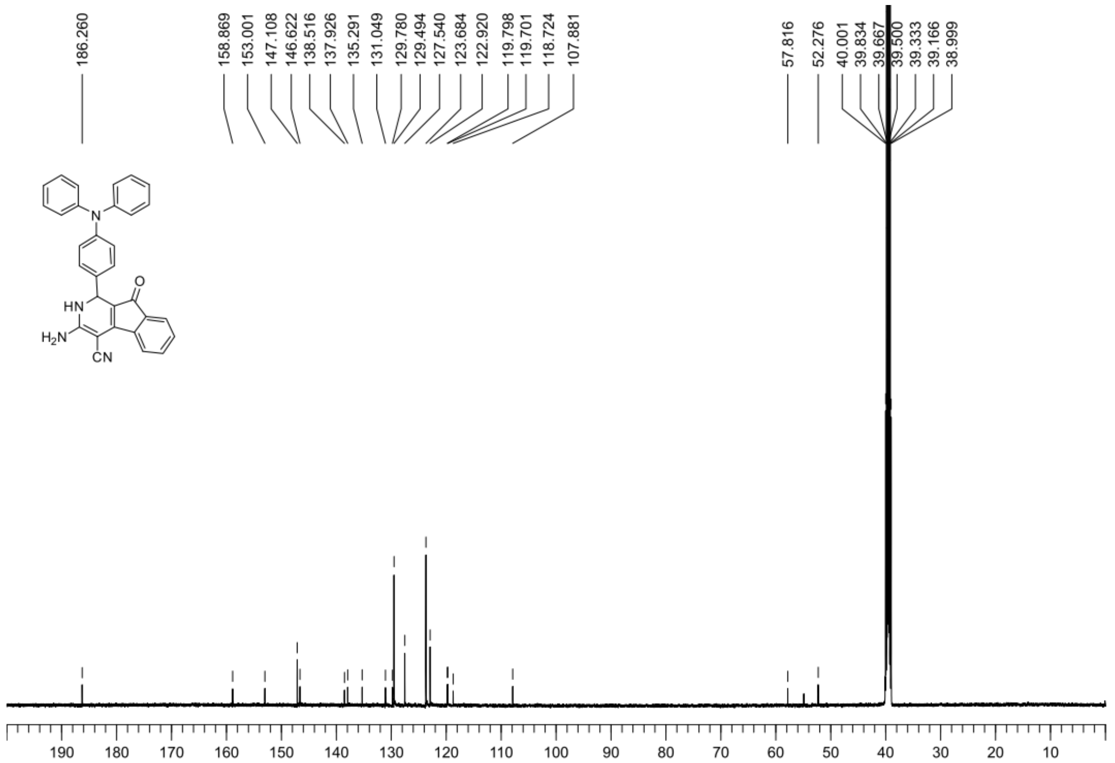

Fig. S3. ^{1}H and ^{13}C NMR spectra of **1c** in DMSO-d_6.

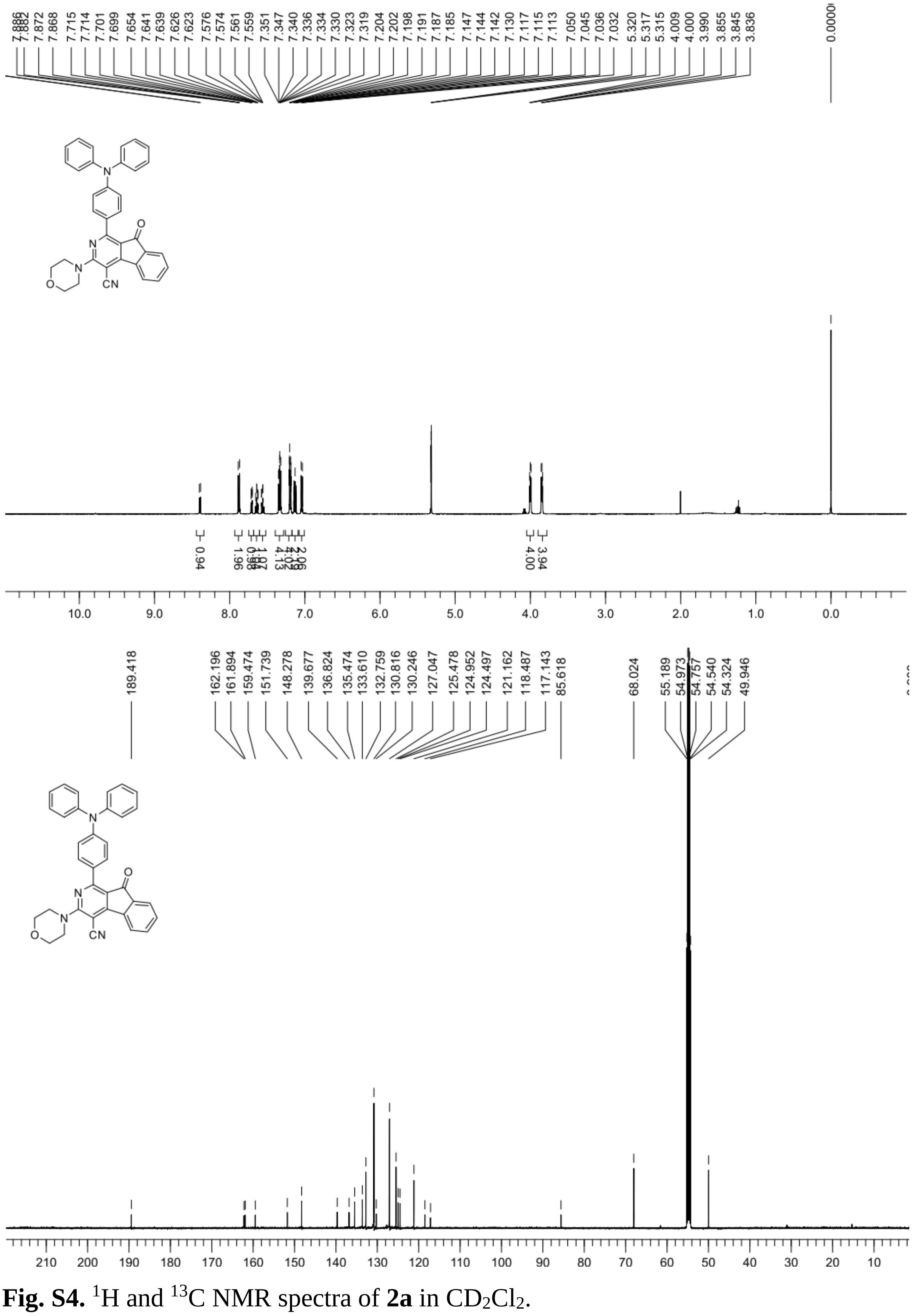

Fig. S4. ^{1}H and ^{13}C NMR spectra of **2a** in CD_2Cl_2.

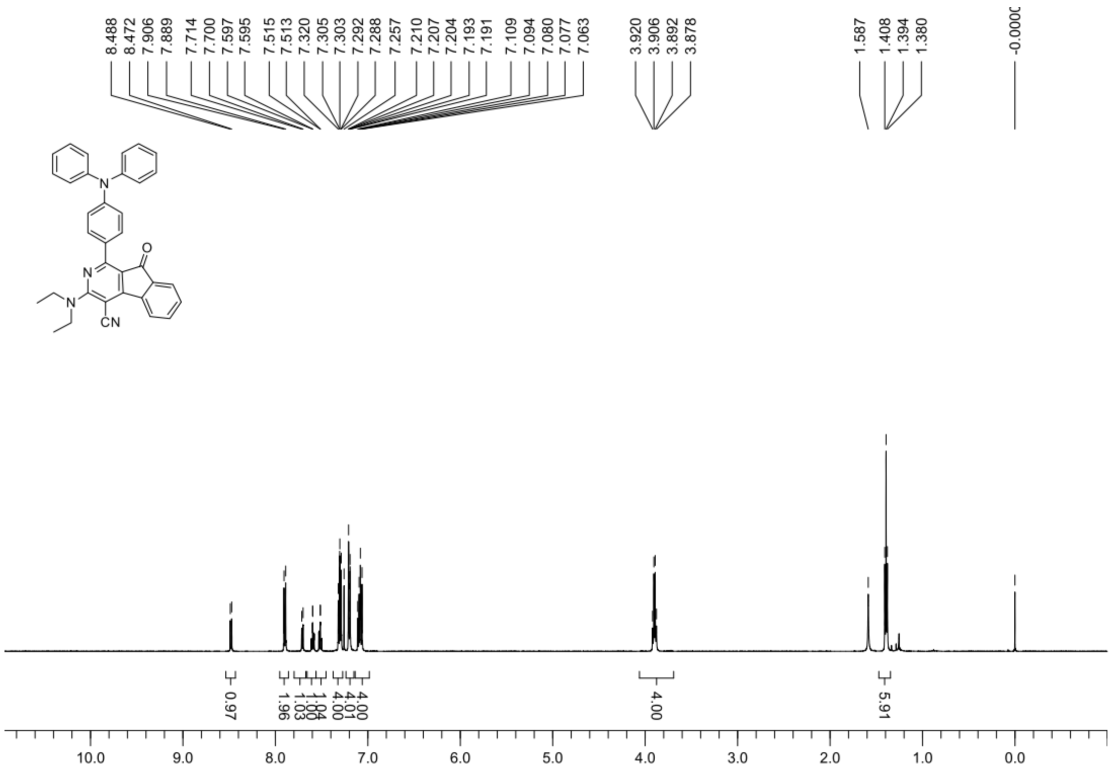

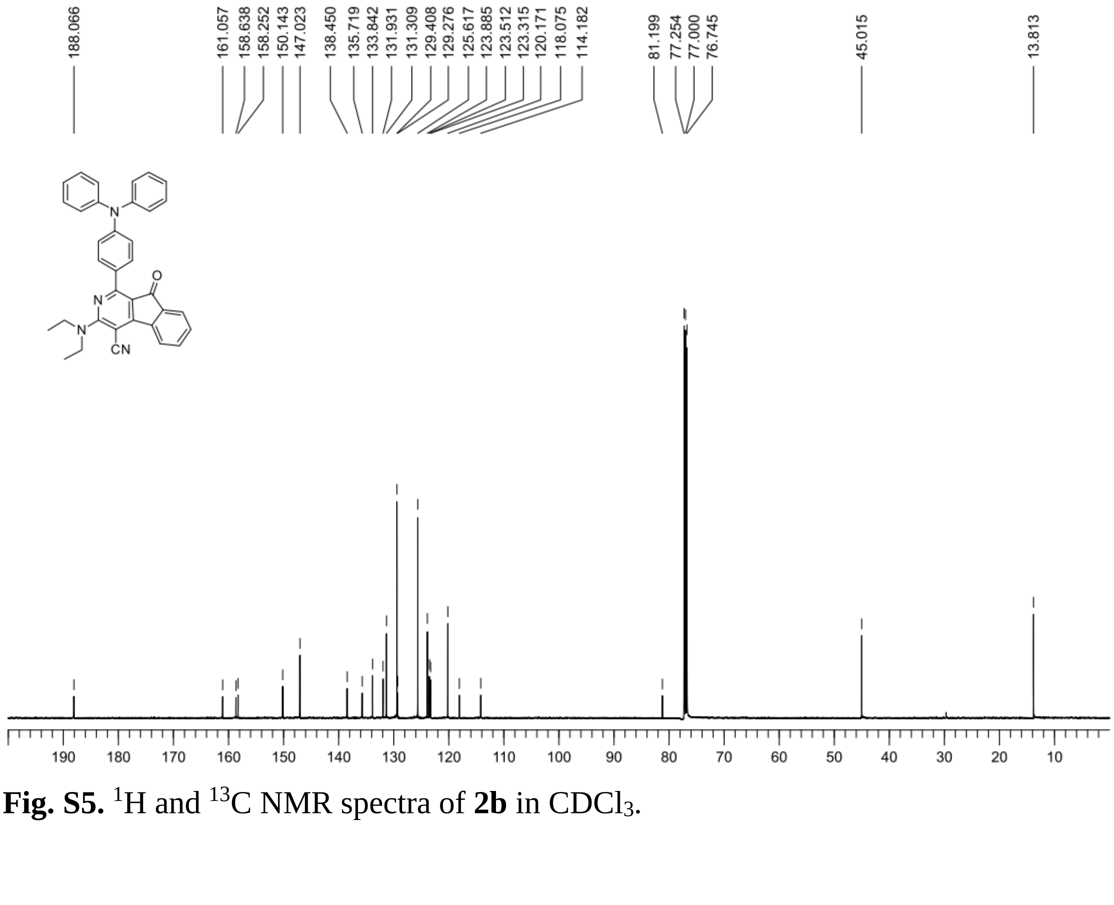

Fig. S5. ^{1}H and ^{13}C NMR spectra of **2b** in $CDCl_3$.

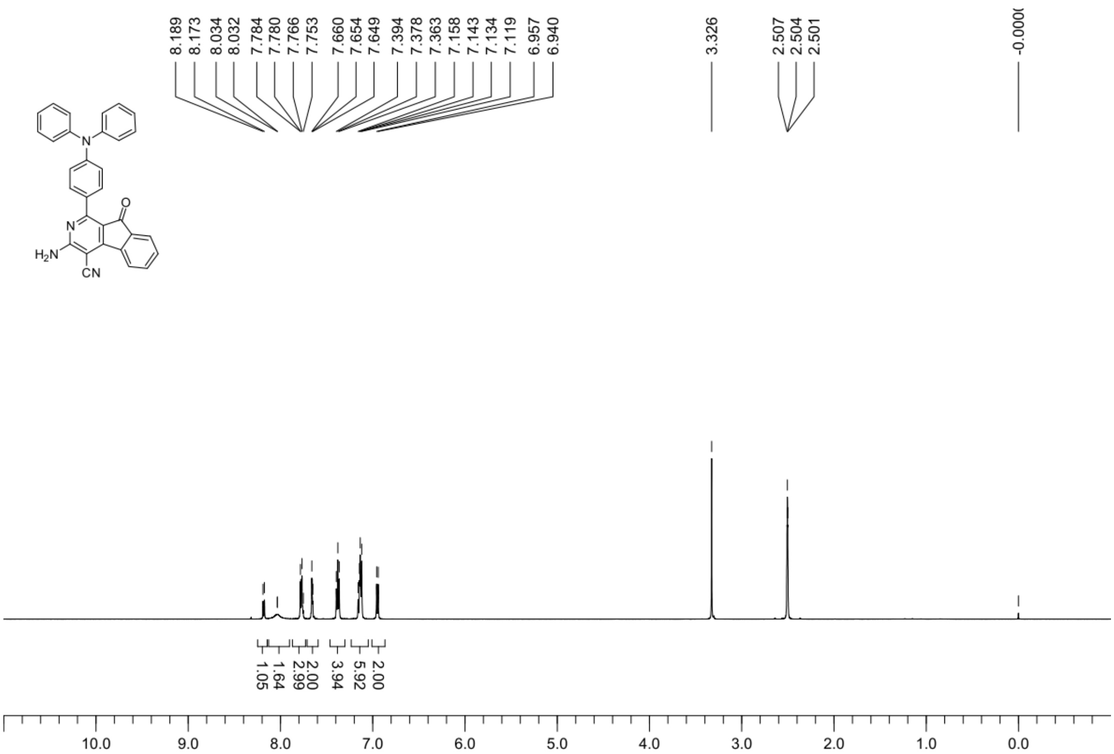

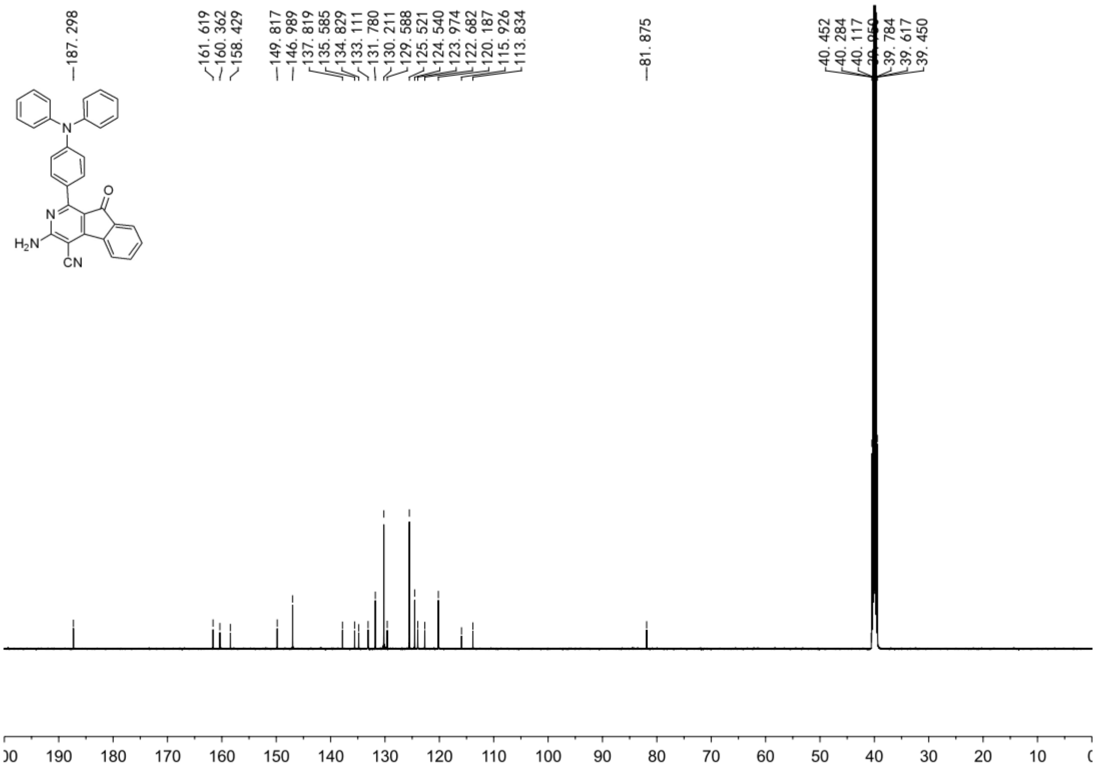

Fig. S6. ^{1}H and ^{13}C NMR spectra of **2c** in DMSO-d_6.

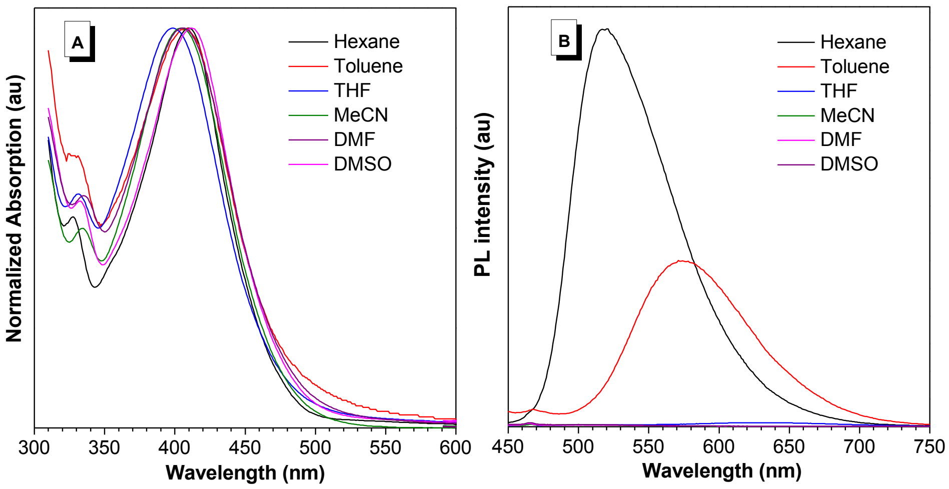

Fig. S7. (A) UV-Vis absorption and (B) PL spectra of **2a** in different solvents. [**2a**] = 10 μM; λ_{ex} = 402 nm.

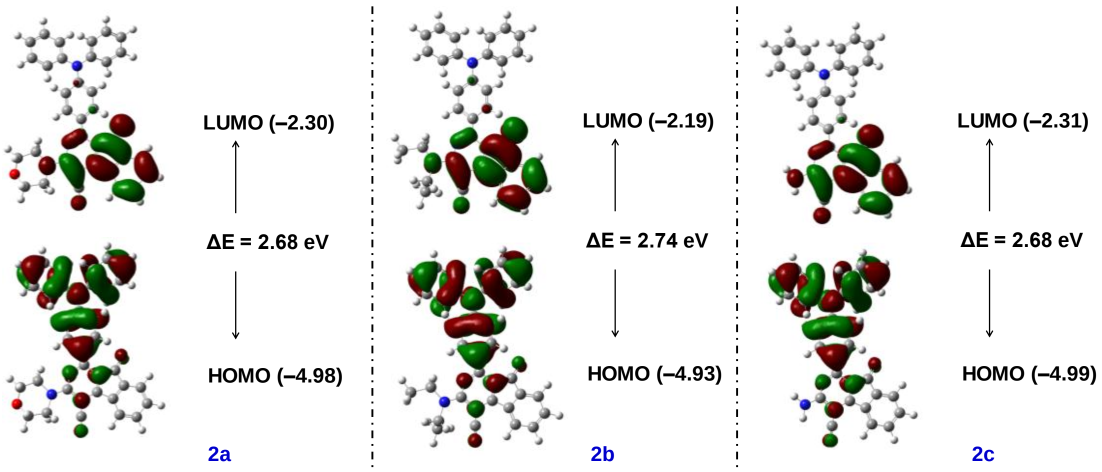

Fig. S8. Molecular orbital amplitude plots of HOMO and LUMO of compound **2**.

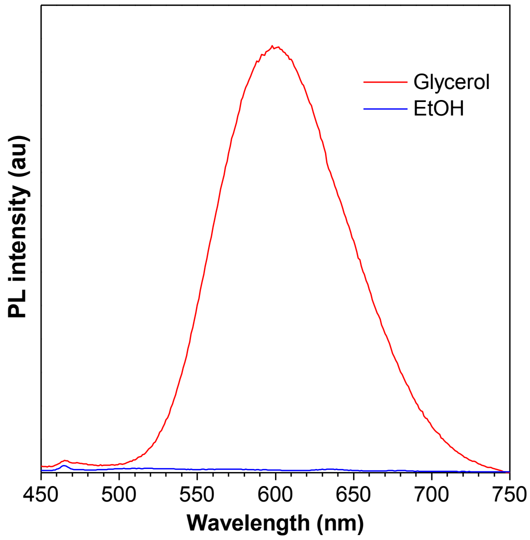

Fig. S9. PL spectra of **2a** in ethanol and glycerol; [**2a**] = 10 μM; λ_{ex} = 402 nm.

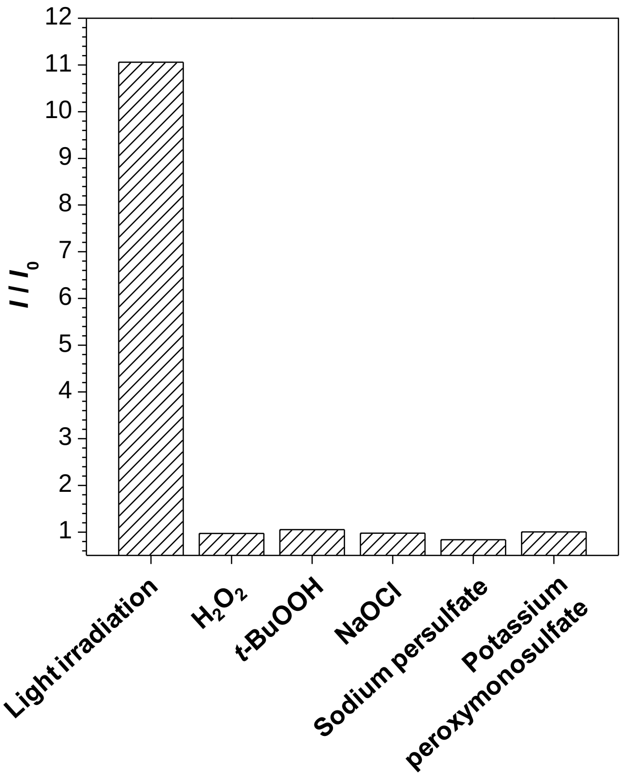

Fig. S10. Fluorescence responses of **1a** (10 μM) to light irradiation with a hand-held UV lamp (365 nm) for 30 min and treated with various oxidative species under dark for 30 min: *t*-BuOOH (1.0 mM), NaOCl (1.0 mM), Sodium persulfate (1.0 mM), Potassium peroxymonosulfate (1.0 mM), and H_2O_2 (1.0 mM) in DMSO/water mixture with 99% water fraction ($\lambda_{ex/em}$ = 409/600 nm), where I_0 and I are the PL intensities of 100 μM **1a** alone and that upon incubation with different species, respectively.

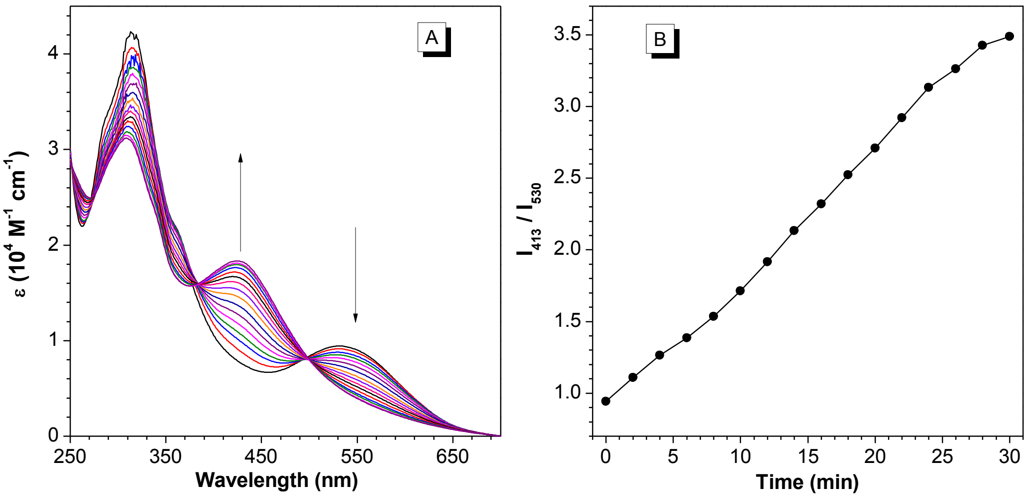

Fig. S11. (A) The UV-Vis spectra of **1a** in DMSO/water mixture with 99% water fraction under irradiation at 365 nm for 0–30 min. (B) Plot of relative UV-Vis absorption intensity (I_{413}/I_{530}) of **1a** under irradiation at 365 nm for 0–30 min.

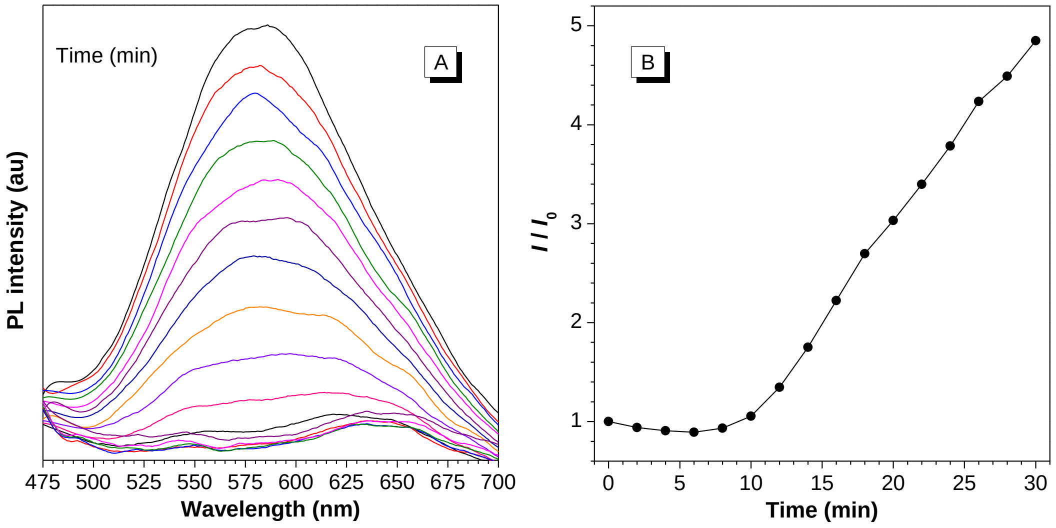

Fig. S12. (A) The PL spectra of **1b** in DMSO/water mixture with 99% water fraction under irradiation at 365 nm for 0–30 min. (B) Plot of relative PL intensity (I/I_0) of **1b** at 581 nm versus the irradiation time.

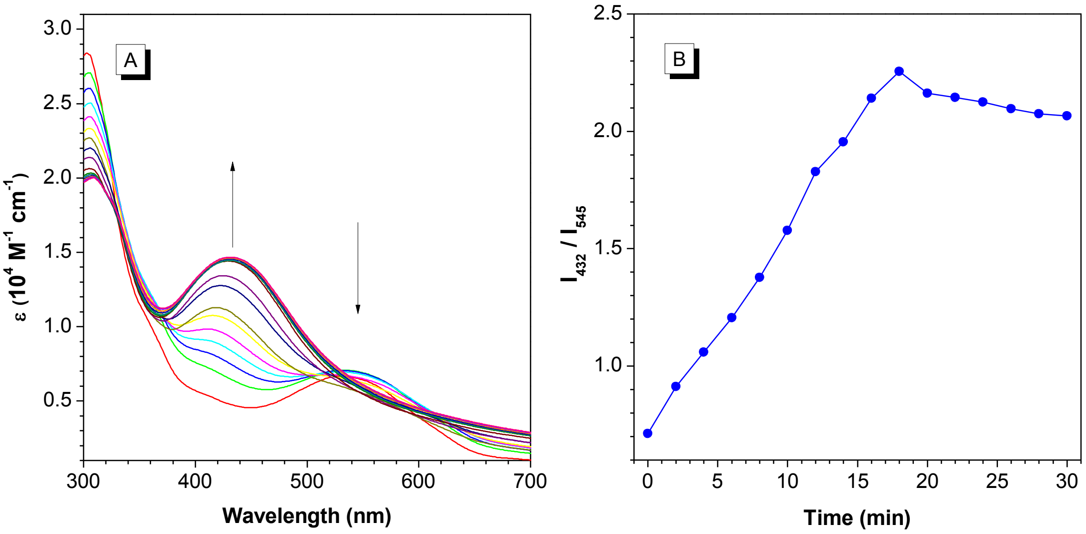

Fig. S13. (A) The UV-Vis spectra of **1b** in DMSO/water mixture with 99% water fraction under irradiation at 365 nm for 0–30 min. (B) Plot of relative UV-Vis absorption intensity (I_{432}/I_{545}) of **1b** under irradiation at 365 nm for 0–20 min.

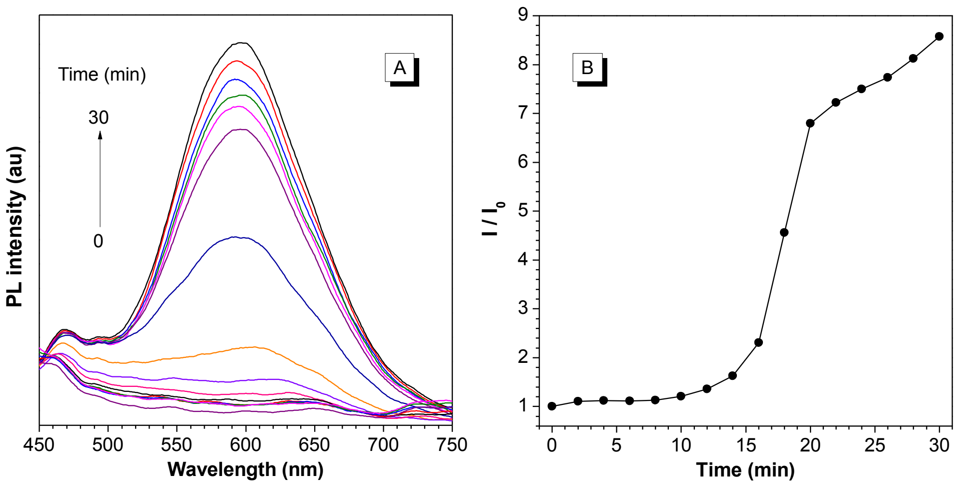

Fig. S14. (A) The PL spectra of **1c** in DMSO/water mixture with 99% water fraction under irradiation at 365 nm for 0–30 min. (B) Plot of relative PL intensity (I/I_0) of **1c** at 581 nm versus the irradiation time.

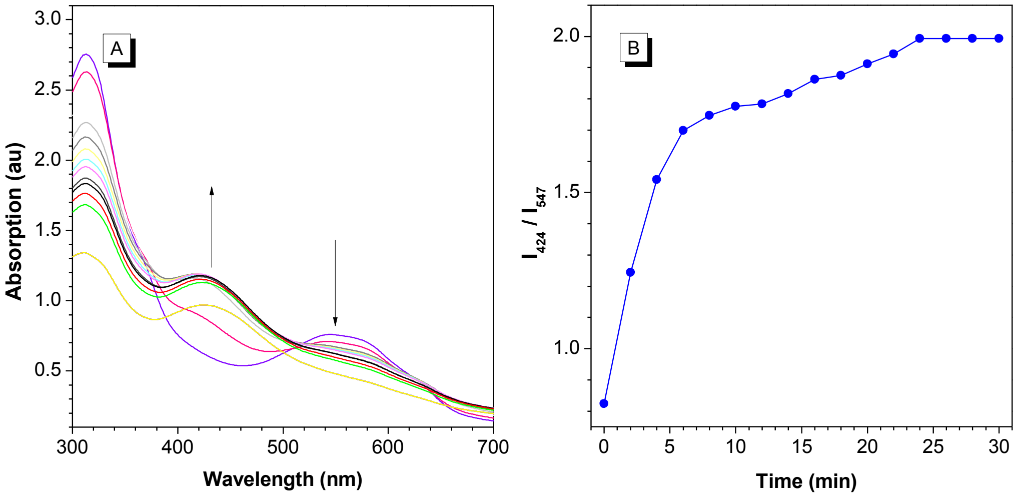

Fig. S15. (A) The UV-Vis spectra of **1c** in DMSO/water mixture with 99% water fraction under irradiation at 365 nm for 0–30 min. (B) Plot of relative UV-Vis absorption intensity (I_{424}/I_{547}) of **1c** under irradiation at 365 nm for 0–20 min.

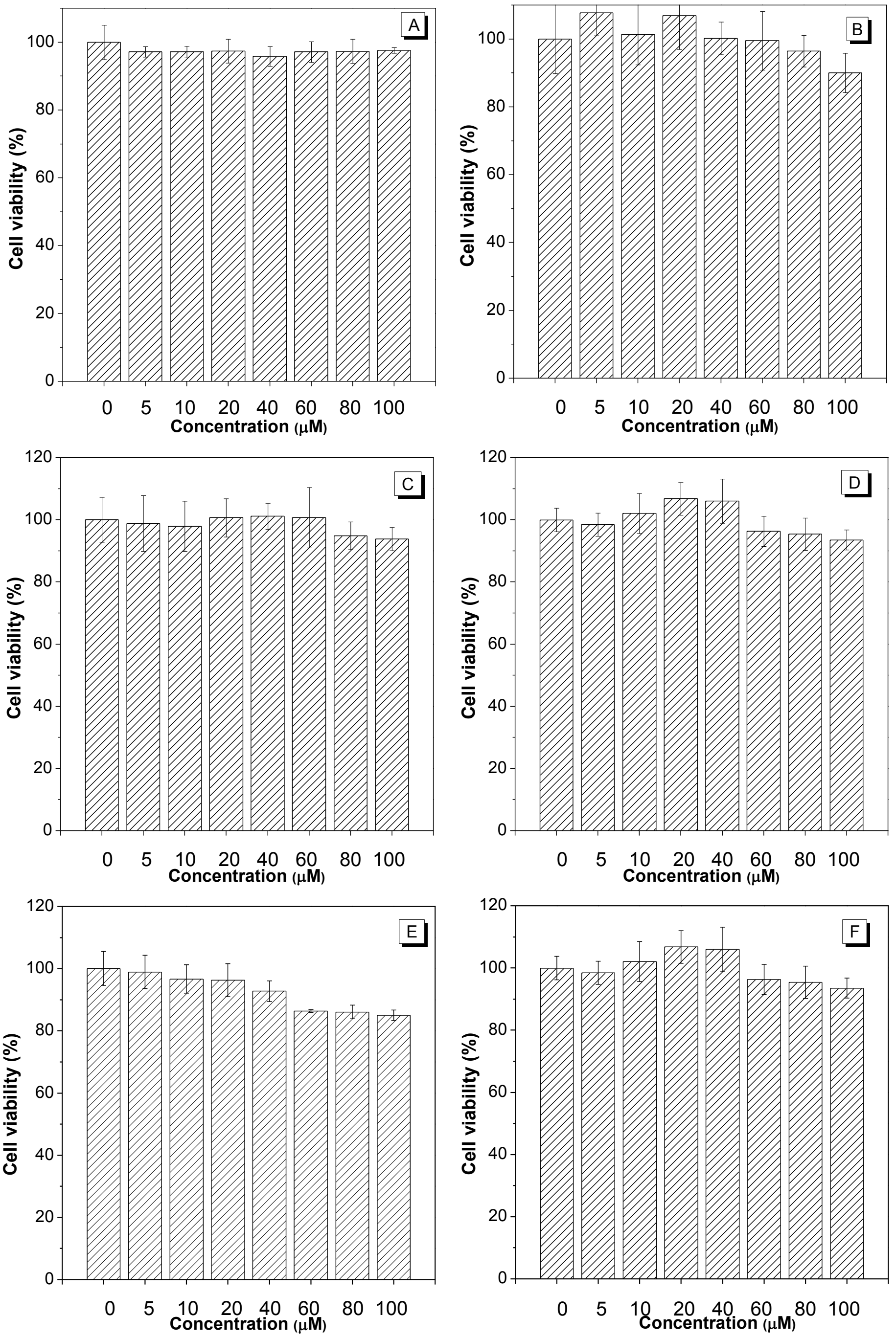

Fig. S16. Cell viabilities of (A) HCC827 lung cancer cells, (C) A549 lung cancer cells, and (E) HLF normal lung cells after incubation with different concentrations of **1a** (0, 5, 10, 20, 40, 60, 80, and 100 μM) for 24 h; Cell viabilities of (B) HCC827 lung cancer cells, (D) A549 lung cancer cells, and (F) HLF normal lung cells after incubation with different concentrations of **2a** (0, 5, 10, 20, 40, 60, 80, and 100 μM) for 24 h.

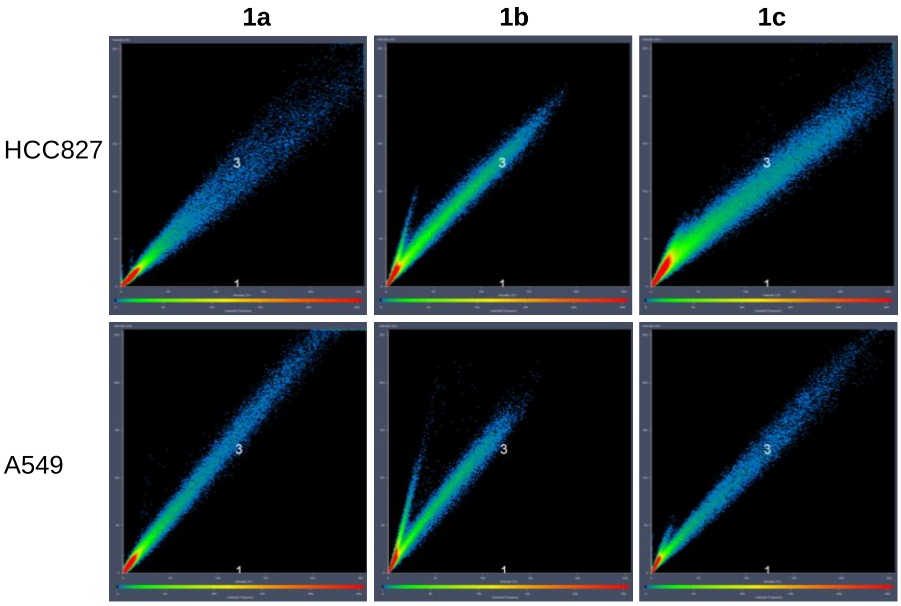

Fig. S17. Intensity correlation plots of photoactivated probes **1** (Y-axis) and BODIPY493/503 Green (X-axis) in HCC827 and A549 cells.

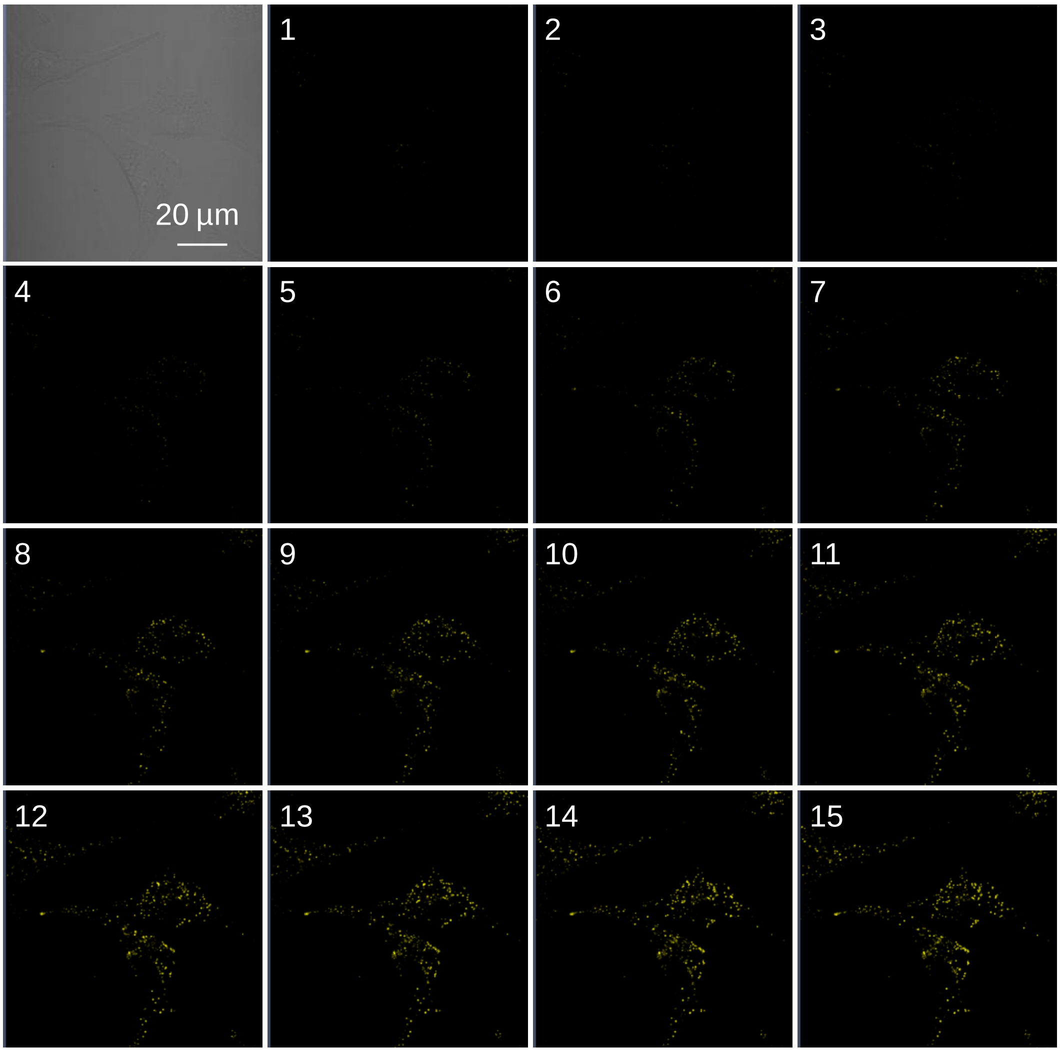

Fig. S18. Bright field and fluorescent images of live A549 cells stained with **1a** taken white light illumination and increasing scans at 405 nm with laser power of 1% (the number of scans are shown in upper left corner and the scanning rate was 22.4 s per frame). All images share the same scale bar of 20 μm.

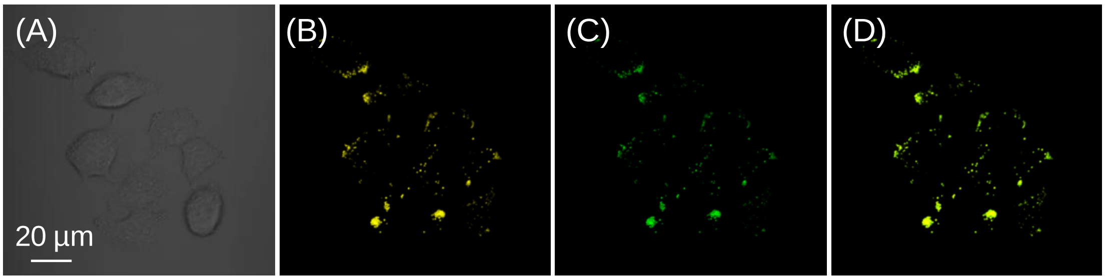

Fig. S19. CLSM images of A549 cells costained with **1a** and BODIPY493/503 Green. (A) Bright field image of A549 cells; (B) fluorescent image from **1a** after photoactivation; (C) fluorescent image from BODIPY493/503 Green; (D) the merged image. All images share the same scale bar of 20 μm. **1a** was photoactivated through continuous scanning at 405 nm (1% laser power) for 5 min.

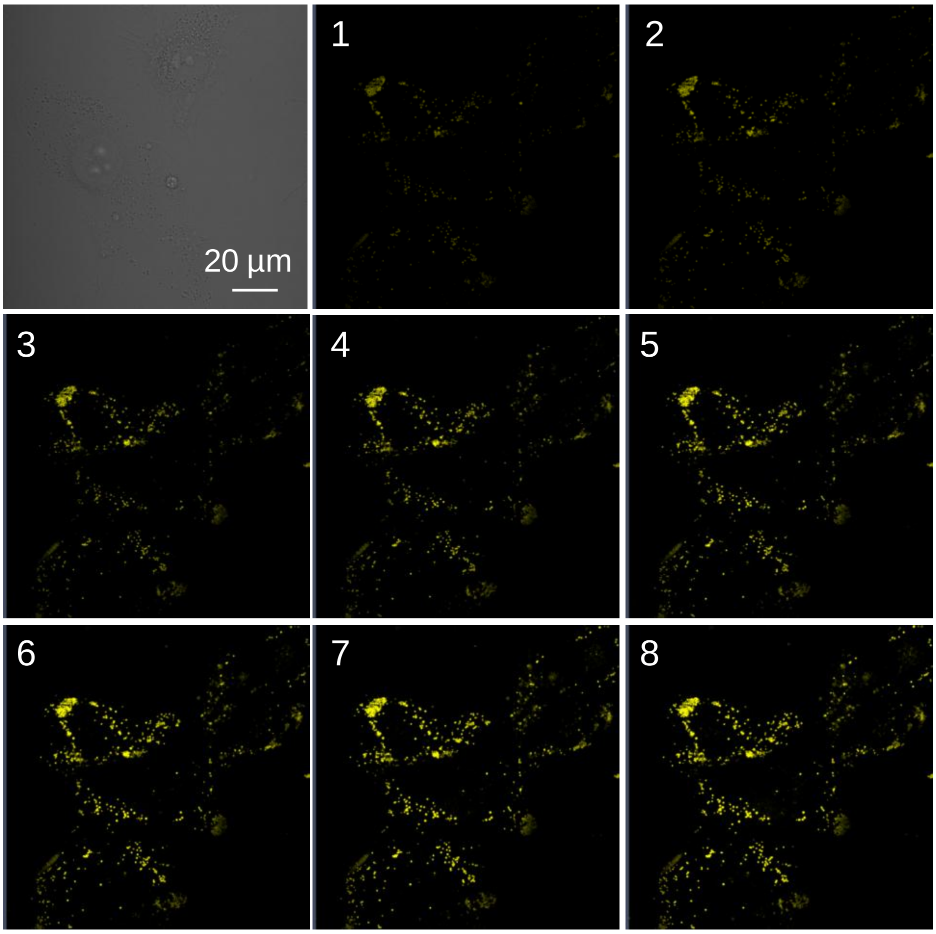

Fig. S20. Bright field and fluorescent images of live HCC827 cells stained with **1b** (20 μM) taken under white light illumination and increasing scans at 405 nm with laser power of 1% (the number of scans are shown in upper left corner and the scanning rate was 22.4 s per frame). All images share the same scale bar of 20 μm.

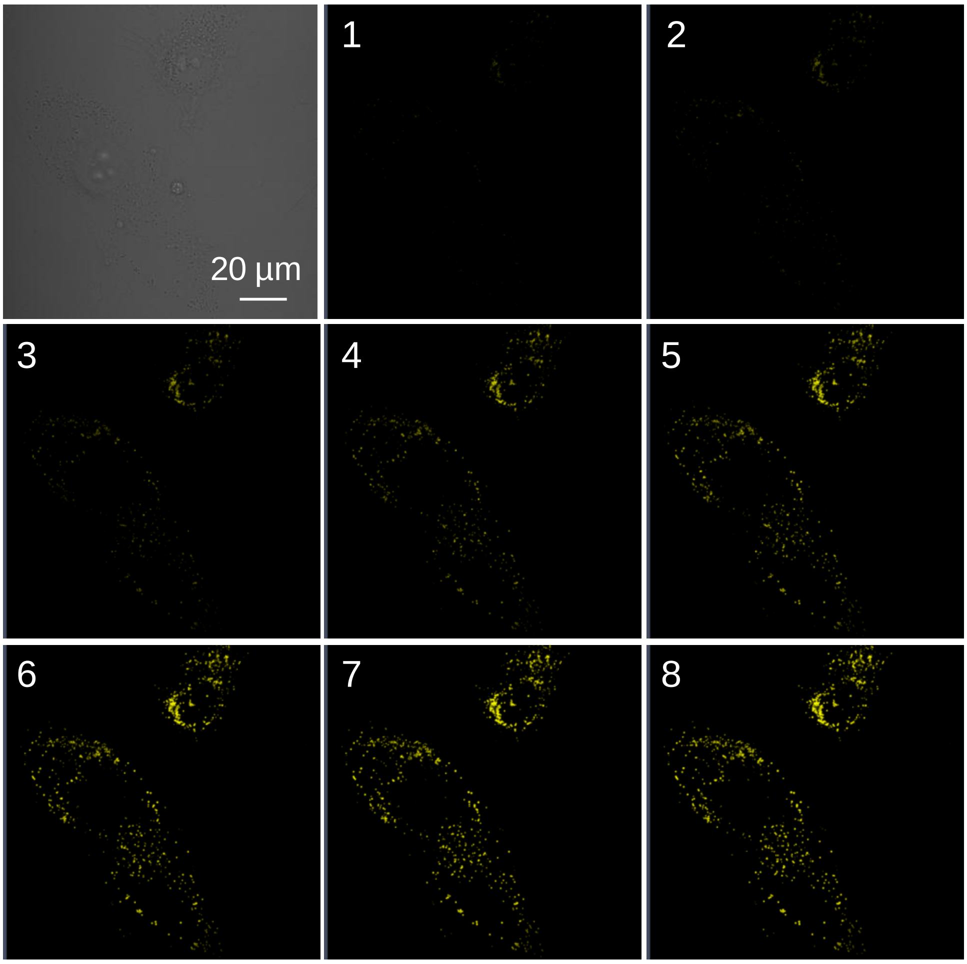

Fig. S21 Bright field and fluorescent images of live HCC827 cells stained with **1c** (20 μM) taken under white light illumination and increasing scans at 405 nm with laser power of 1% (the number of scans are shown in upper left corner and the scanning rate was 22.4 s per frame). All images share the same scale bar of 20 μm.

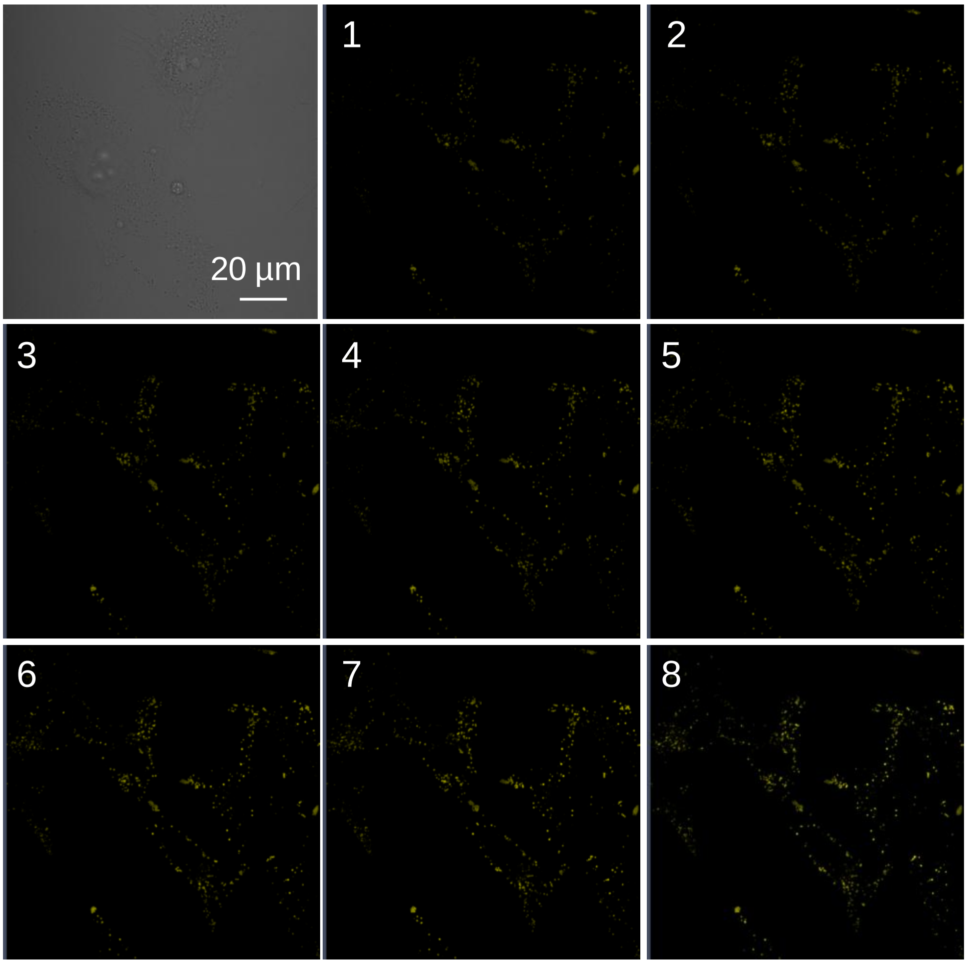

Fig. S22. Bright field and fluorescent images of live A549 cells stained with **1b** (20 µM) taken under white light illumination and increasing scans at 405 nm with laser power of 1% (the number of scans are shown in upper left corner and the scanning rate was 22.4 s per frame). All images share the same scale bar of 20 µm.

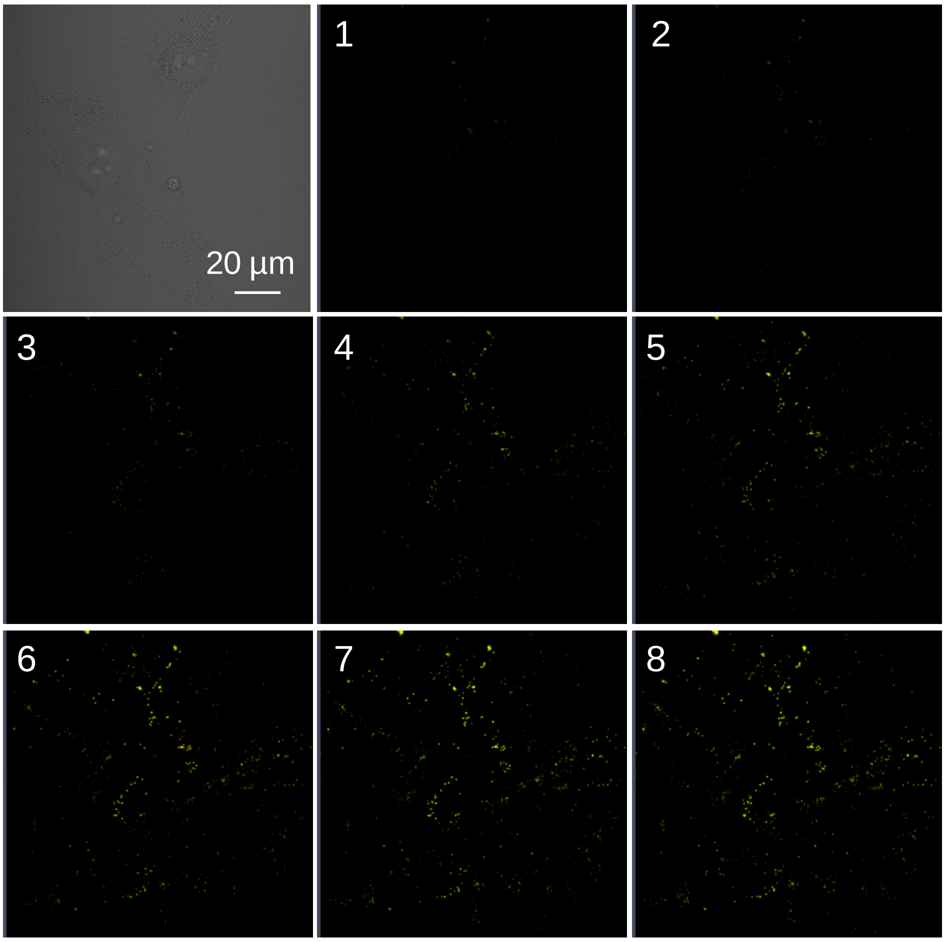

Fig. S23. Bright field and fluorescent images of live A549 cells stained with **1c** (20 µM) taken under white light illumination and increasing scans at 405 nm with laser power of 1% (the number of scans are shown in upper left corner and the scanning rate was 22.4 s per frame). All images share the same scale bar of 20 µm.

(1) Y. Shang, Y. Wen, S. Li, S. Du, X. He, L. Cai, Y. Li, L. Yang, H. Gao and Y. Song, *J. Am. Chem. Soc.*, 2007, **129**, 11674-11675.
